# PGK1 Suppresses CD8^+^ T Cell‐Mediated Antitumor Immunity Through CCL2/CCR2/Tumor‐Associated Macrophages Axis in Hepatocellular Carcinoma

**DOI:** 10.1002/advs.75801

**Published:** 2026-05-25

**Authors:** Xi Liu, Jianpeng Liu, Zhihao Zhang, Xizhi Yu, Junjie Qian, Limin Ding, Qinchuan Wu, Zhe Yang, Xing‐Yu Luo, Xinjiang Ding, Rong Su, Xiaobo Yu, Yiting Qiao, Shengyong Yin, Haiyang Xie, Lin Zhou, Yuanxing Liu, Shusen Zheng

**Affiliations:** ^1^ Division of Hepatobiliary and Pancreatic Surgery Department of Surgery The First Affiliated Hospital Zhejiang University School of Medicine Hangzhou China; ^2^ NHC Key Laboratory of Combined Multi‐Organ Transplantation Key Laboratory of Organ Transplantation Hangzhou Zhejiang China; ^3^ Institute of Organ Transplantation Zhejiang University Hangzhou China; ^4^ State Key Laboratory for Diagnosis and Treatment of Infectious Diseases National Clinical Research Center for Infectious Diseases Hangzhou China; ^5^ Department of Hepaticobiliarypancreatic and Minimally Invasive Surgery Zhejiang Provincial People's Hospital People's Hospital of Hangzhou Medical College Hangzhou China; ^6^ Department of Hepatobiliary and Pancreatic Surgery The Second Affiliated Hospital Zhejiang University School of Medicine Hangzhou China; ^7^ Department of Breast Surgery The First Affiliated Hospital Zhejiang University School of Medicine Hangzhou China; ^8^ Department of Hepatobiliary and Pancreatic Surgery Shulan (Hangzhou) Hospital Affiliated to Zhejiang Shuren University Shulan International Medical College Hangzhou China

**Keywords:** CCL2, CD8^+^ T cell, hepatocellular carcinoma, PGK1, tumor‐associated macrophages

## Abstract

Patients with advanced hepatocellular carcinoma (HCC) have a poor prognosis and few effective treatments. The highly immunosuppressive tumor microenvironment and multifaceted immune escape mechanisms in HCC profoundly restrict the clinical efficacy of immune checkpoint inhibitors. Phosphoglycerate kinase 1 (PGK1) is established as an oncogenic driver in malignant tumor progression. However, its role in modulating tumor immunity remains elusive. This study found that HCC cell‐intrinsic PGK1 contributed to tumor progression through inhibiting CD8^+^ T cell‐mediated antitumor immunity. Then, in vivo and in vitro results demonstrated that tumor‐associated macrophages (TAMs) function as critical mediators in PGK1‐driven immunosuppression. Furthermore, PGK1 promoted the recruitment and M2 polarization of TAMs through upregulation of C‐C motif chemokine ligand 2 (CCL2) expression. Blockade of CCL2 significantly attenuated PGK1‐induced infiltration of C‐C motif chemokine receptor 2 (CCR2)^+^ TAMs and inhibited M2 polarization, while reversing the impaired infiltration and function of CD8^+^ T cells. This work further demonstrated that PGK1 enhanced CCL2 secretion through activating the AKT/GSK3β/β‐catenin pathway in HCC cells. In vivo experiments revealed that the combination of PGK1 inhibitor and immunotherapy elicited potent antitumor efficacy. Taken together, this study highlighted PGK1 as a pivotal regulator of tumor immunobiology and provided novel insights into combination immunotherapy for HCC.

## Graphical abstract

PGK1 in tumor cells upregulates CCL2 expression through activation of the AKT/GSK‐3β/β‐catenin signaling axis, thereby promoting the recruitment and M2 polarization of TAMs and ultimately impairing the infiltration and activation of CD8^+^ T cells within the HCC tumor microenvironment.

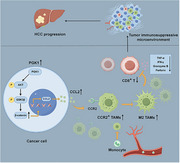

## 1 Introduction

Hepatocellular carcinoma (HCC) ranks as the sixth most prevalent cancer globally and represents the third leading cause of cancer‐related mortality [1, 2]. Advanced HCC accounts for 70%–80% of all cases. The tyrosine kinase inhibitors (TKIs) sorafenib and lenvatinib were once approved as the first‐line therapy for advanced HCC, but they had limited effects on survival [3, 4]. The emergence of immunotherapy in recent years has provided new hope for improving survival in various solid tumors, including HCC [5, 6]. Immune checkpoint inhibitors (ICIs), particularly those targeting programmed death‐1 (PD‐1) and programmed death‐ligand 1 (PD‐L1), have become key treatments for advanced HCC [7, 8]. As immune checkpoint monotherapies fail to induce a satisfactory overall response in HCC, highlighting an urgent need to develop more innovative combination strategies that restore or augment sensitivity to drug‐resistant tumors [5, 9].

The tumor microenvironment (TME) plays a pivotal role in tumorigenesis and progression, primarily composed of tumor cells, immune cells, cytokines, and extracellular matrix [10]. The TME in HCC is mainly dominated by immunosuppressive cells and signals, fostering a tolerant and immunosuppressive microenvironment niche [5, 11]. Therefore, a good understanding of immune evasion mechanisms within TME in HCC is essential to improving the efficacy of immunotherapy. Tumor‐associated macrophages (TAMs) are the most abundant immune cells in the HCC microenvironment, exhibiting high heterogeneity and functional plasticity, which contribute to immune evasion, immunotherapy resistance, tumor growth, and metastasis [12, 13]. In addition, tumor cells can recruit macrophages via specific chemokines and cytokines, reprogramming them into an immunosuppressive M2 phenotype that suppresses T cell function [14, 15]. Therefore, further elucidating the crosstalk between tumor cells and TAMs will facilitate the development of combination immunotherapies.

Phosphoglycerate kinase 1 (PGK1) is a key enzyme in the aerobic glycolysis process that catalyzes the reversible reaction of 1,3‐bisphosphoglycerate to 3‐phosphoglycerate. It functions not only as a metabolic enzyme but also as a protein kinase, playing a role as an oncogene through the regulation of angiogenesis, mitochondrial metabolism, epithelial‐mesenchymal transformation, and autophagy [16, 17, 18, 19]. PGK1 is abnormally overexpressed in a variety of human tumors and is correlated with poor prognosis [20, 21, 22]. Most studies currently focus on the effects of PGK1 post‐translational modifications and enzyme activity on tumorigenesis and cancer development. However, the role of PGK1 in the HCC TME has received little attention [20, 23, 24].

In this study, we demonstrated that PGK1 drives HCC progression by promoting TAMs recruitment and M2 polarization, thereby suppressing the infiltration and activation of CD8^+^ T cells in the TME. Mechanistically, this effect was mediated by PGK1 in HCC cells through activation of the AKT/GSK‐3β/β‐catenin signaling pathway, leading to upregulation of C‐C motif chemokine ligand 2 (CCL2) and subsequent establishment of an immunosuppressive microenvironment. Pharmacological targeting of PGK1 with the selective inhibitor NG 52 significantly enhanced the efficacy of anti‐PD‐L1 antibody in orthotopic HCC models. These findings suggested tumor cell‐intrinsic PGK1 represents a promising biomarker and therapeutic target to enhance immunotherapy efficacy in HCC.

## 2 Results

### 2.1 High Expression of PGK1 Correlates With Poor Prognosis in HCC

**FIGURE 1:**
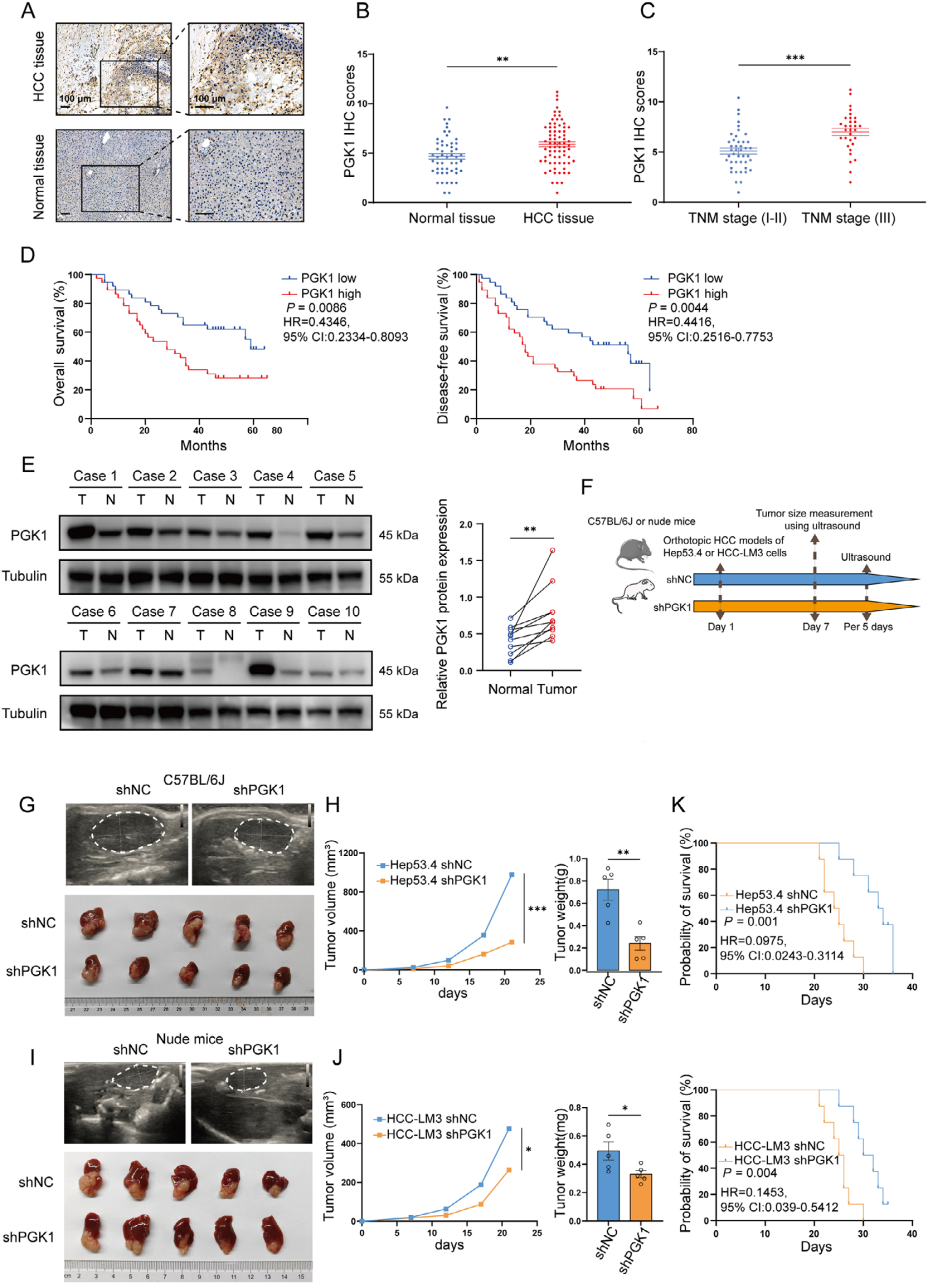
High expression of PGK1 correlates with poor prognosis in HCC. (A) Representative IHC staining pictures of PGK1 in HCC tumor tissues and normal tissues (left panel, 200x magnification; right panel, 400x magnification). (B) IHC scores of PGK1 staining in HCC tumor tissues (*n* = 74) and normal tissues (*n* = 52). (C) Comparison of PGK1 expression between early stage HCC (TNM stage I/ II, *n* = 43) and advanced stage HCC (TNM stage III, *n* = 31). (D) Overall survival analysis (left) and disease‐free survival analysis (right) of HCC patients with high PGK1 expression (IHC staining scores ≥ 6, *n* = 37) and patients with low PGK1 expression (IHC scores < 6, *n* = 37). (E) Western blot analysis of PGK1 expression in 10 pairs of human HCC tumor tissues and adjacent normal tissues. (F) Schematic illustration of Hep53.4 and HCC‐LM3 orthotopic HCC models in mice. (G) Representative ultrasound images for shPGK1 and shNC Hep53.4 orthotopic tumors in C57BL/6J mice (upper) and gross images of orthotopic liver tumors (lower) (*n* = 5). (H) Tumor volume growth curves (left) and tumor weight (right) of shPGK1 and shNC Hep53.4 orthotopic HCC models (*n* = 5). (I) Representative ultrasound images for shPGK1 and shNC HCC‐LM3 orthotopic tumors in nude mice (upper) and gross images of orthotopic liver tumors (lower) (*n* = 5). (J) Tumor volume growth curves (left) and tumor weight (right) of shPGK1 and shNC HCC‐LM3 orthotopic HCC models (*n* = 5). (K) Comparison of survival between shPGK1 and shNC orthotopic HCC models in C57BL/6J (upper) and nude mice (lower) (*n* = 8). HR, hazard ratio. 95% CI, 95% confidence interval. Data were presented as mean ± SEM. ns, no significant difference. ^*^ *p* < 0.05; ^**^ *p* < 0.01; ^***^ *p* < 0.001; ^****^ *p* < 0.0001.

To evaluate underlying associations between PGK1 and HCC progression, we first explored the differential expression of PGK1 in HCC tissues and adjacent normal tissues using data from The Cancer Genome Atlas (TCGA). We found that PGK1 showed obviously higher expression in HCC tumor tissues than in normal tissues (Figure S1A). Furthermore, patients with elevated levels of PGK1 had a significantly poorer survival (Figure S1B). We then performed immunohistochemistry (IHC) with tissue sections derived from HCC patients to further investigate the expression of PGK1 and its clinical relevance in HCC. Consistent with the results of the TCGA data, our results confirmed the high pgk1 expression levels in HCC tumor tissues, and poor outcomes were found in those with high pgk1 expression (Figure 1A–D). In addition, Western blot analysis of 10 matched tissue pairs showed significantly higher PGK1 expression in tumor tissues than in corresponding normal tissues (Figure 1E). We next sought to validate these findings in vivo. we injected stable PGK1 knockdown (shPGK1) or control (shNC) HCC‐LM3 and Hep53.4 cells separately into the liver of immune competent C57BL/6J mice and immune compromised nude mice (Figure 1F). The orthotopic HCC models showed PGK1 knockdown could significantly suppress tumor progression in both C57BL/6J mice and nude mice (Figure 1G–J). This observation was further corroborated in Hepa1‐6 and PLC/PRF/5 orthotopic tumor models independently (Figure S1C–F). Moreover, PGK1 knockdown significantly improved the survival rate of tumor‐bearing mice (Figure 1K). These results demonstrated that high PGK1 expression is closely associated with poor prognosis in HCC.

### 2.2 PGK1 Promotes Tumor Progression by Inducing Immunosuppression Through the Attenuation of CD8^+^ T Cell Responses

**FIGURE 2:**
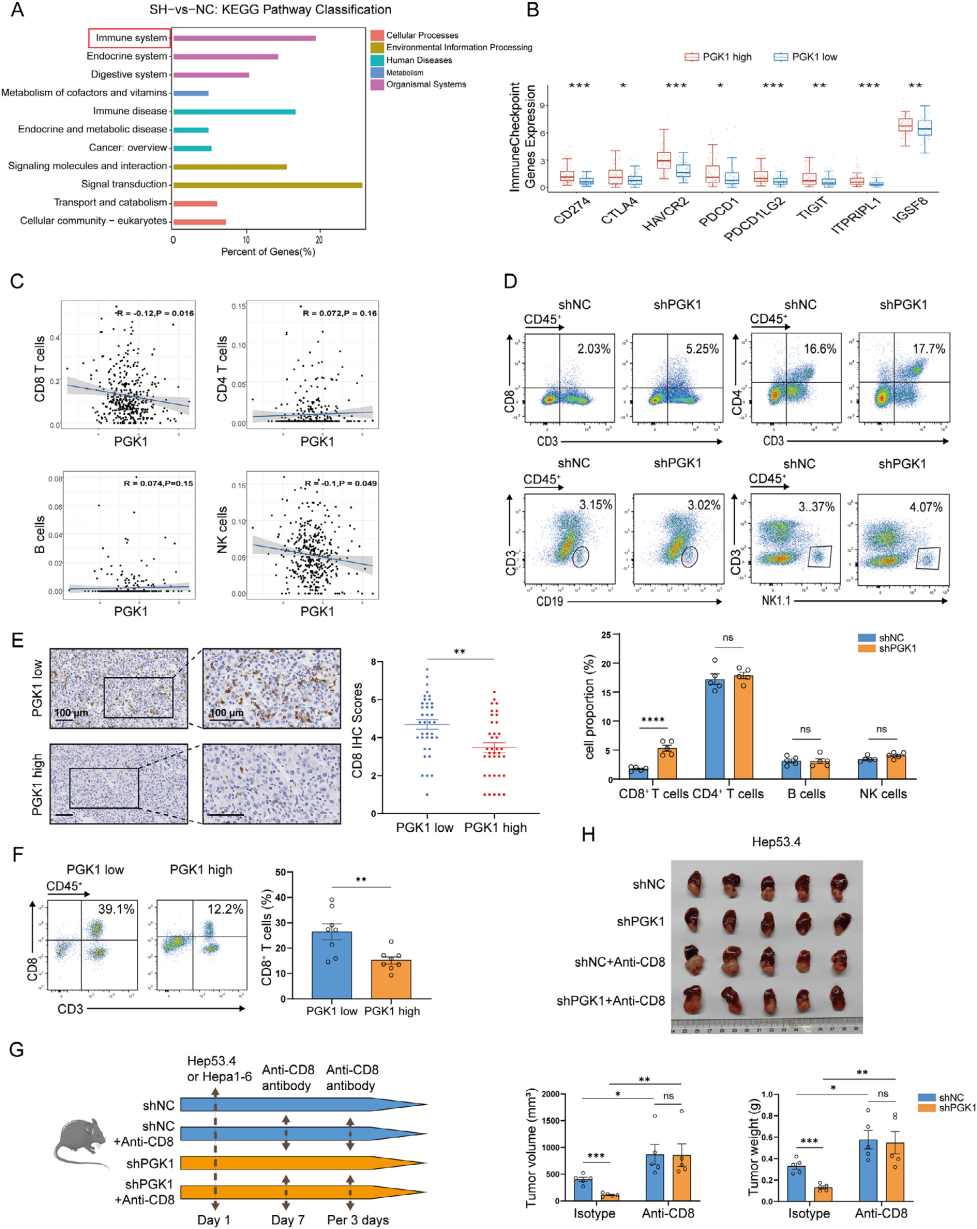
PGK1 promotes tumor progression by inducing immunosuppression through the attenuation of CD8^+^ T cell responses. (A) KEGG pathway classification of differentially expressed genes in the transcriptome sequencing. (B) Relative expression levels of eight key immune‐regulation‐related genes between high PGK1 expression and low PGK1 expression in the TCGA HCC cohort. (C) Analysis of the correlation between PGK1 expression and the infiltration of CD8^+^ T cells, CD4^+^ T cells, B cells, and NK cells in the TCGA database. (D) Flow cytometry analysis of the infiltration levels of CD8^+^ T cells, CD4^+^ T cells, B cells, and NK cells in Hep53.4 orthotopic HCC models in C57BL/6J mice (*n* = 5). (E) Representative IHC staining pictures of CD8 in PGK1 high expression samples (*n* = 37) and PGK1 low expression samples *n* = 37) (left, 200x and 400x magnification) and comparison of the IHC scores of CD8 (right). (F) Flow cytometry analysis of the infiltration of CD8^+^ T cells in tumor samples from HCC patients (*n* = 8). (G) Schematic illustration of orthotopic HCC models in C57BL/6J mice using Hep53.4 or Hepa1‐6 cells, along with CD8^+^ T cell depletion. (H) Gross images of shPGK1 and shNC Hep53.4 orthotopic tumors with or without CD8^+^ T cells depletion (upper), and comparison of tumor volume and tumor weight (lower) (*n* = 5). Data were presented as mean ± SEM. ns, no significant difference. ^*^ *p* < 0.05; ^**^ *p* < 0.01; ^***^ *p* < 0.001; ^****^ *p* < 0.0001.

Given that PGK1 knockdown exerted a substantially greater tumor‐suppressive effect in the orthotopic C57BL/6J than in nude mice, with tumor growth inhibition rates (calculated based on tumor weight) of 66.6% and 33.2%, respectively, we further performed two‐way ANOVA, which revealed a significant interaction between mouse strain and PGK1 knockdown treatment (*p* = 0.0276). These results indicated that the anti‐tumor effect of PGK1 knockdown was more pronounced in immunocompetent mice. Based on the above findings, we speculated that PGK1 in HCC cells not only drives intrinsic tumor growth but also plays an important role in suppressing anti‐tumor immunity. Our transcriptomic data revealed that gene alterations in the immune system were highly pronounced following PGK1 knockdown (Figure 2A). The analysis of the TCGA database also indicates that the expression levels of eight key immune‐regulation‐related genes are significantly higher in the PGK1 high expression group than those in the low expression group (Figure 2B). Next, we explored the association between tumor PGK1 and tumor‐infiltrating immune cells within TME in HCC by analyzing TCGA data. The analysis showed that PGK1 expression was significantly negatively correlated with infiltrating levels of CD8^+^ T cells and NK cells, while no significant associations were found with other immune cells, including CD4^+^ T cells, B cells, regulatory T cells (Tregs), and Dendritic cells (DCs) (Figure 2C and Figure S2A). Then we analyzed the changes of immune cells quantified in the orthotopic HCC models of C57BL/6J mice by flow cytometry analyses. Consistently, compared with shNC Hep53.4 orthotopic tumors, shPGK1 Hep53.4 orthotopic tumors had more infiltrating CD8^+^ T cells (Figure 2D and Figure S2B). However, there was no difference in the infiltration of NK cells. Similar results were obtained in the Hepa1‐6 orthotopic HCC models (Figure S2C). IHC and flow cytometry analyses of tumor samples from HCC patients also confirmed the negative correlation between tumor PGK1 expression and CD8^+^ T cells density (Figure 2E,F). Given the central role of cytotoxic CD8^+^ T cells in antitumor immunity, we next investigated whether the protumor effects of PGK1 were mediated by suppressing CD8^+^ T cell immunity. We depleted CD8^+^ T cells by intraperitoneal injection of anti‐CD8 monoclonal antibody in tumor‐bearing mice (Figure 2G). Notably, administration with anti‐CD8 antibody dramatically promoted tumor growth both in the shPGK1 and shNC groups (Figure 2H and Figure S2D). However, the depletion of CD8^+^ T cells in vivo resulted in similar tumor size and weight between shPGK1 and shNC tumors, indicating that inhibiting CD8^+^ T cells abolished the tumor suppressive effect elicited by PGK1 knockdown. Collectively, these results suggested that PGK1 contributes to tumor progression through suppressing CD8^+^ T cell‐mediated antitumor immunity.

### 2.3 TAMs Mediate the Suppression Effects of PGK1 on CD8^+^ T Cells

**FIGURE 3:**
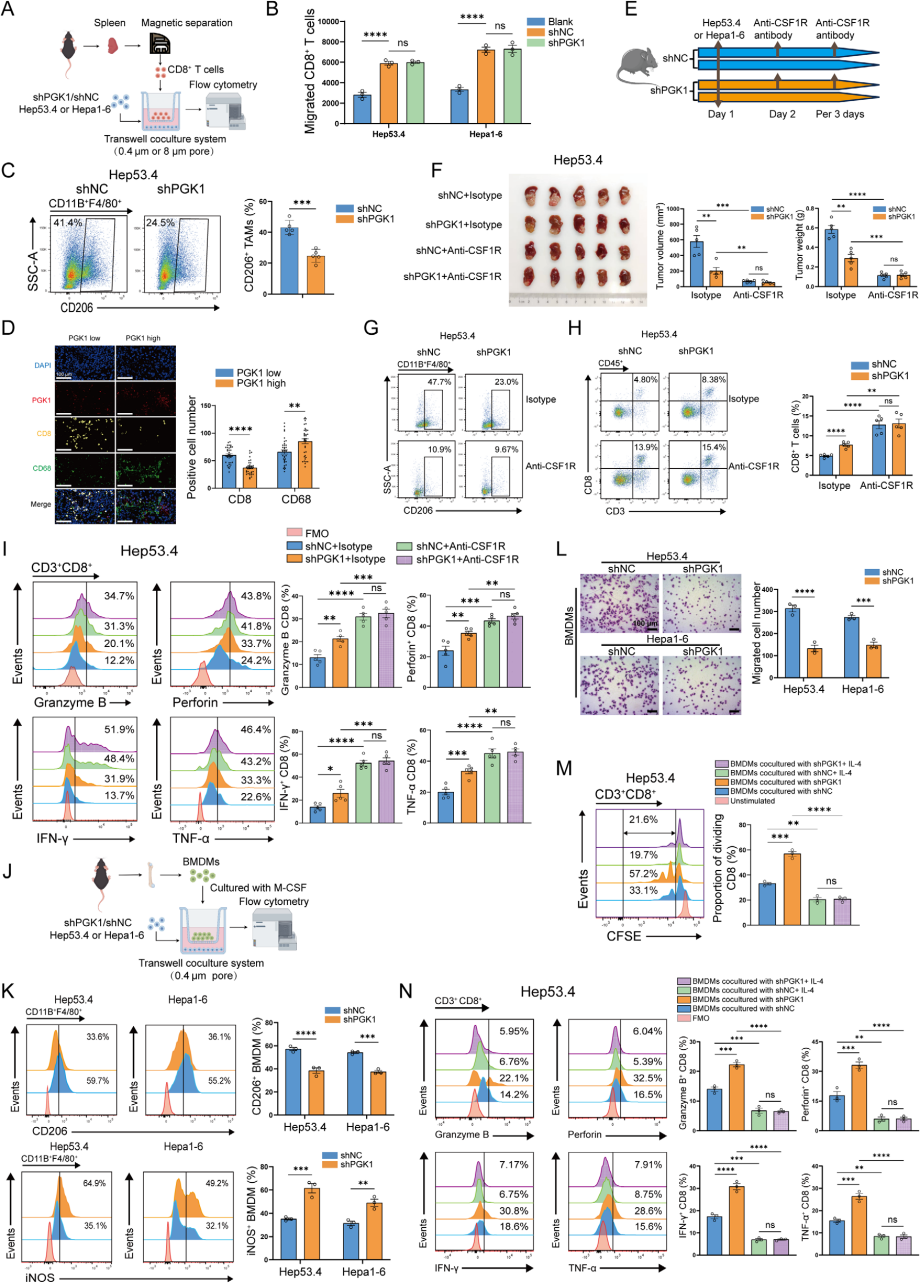
TAMs mediated the suppression effects of PGK1 on CD8^+^ T cells. (A) Schematic illustration of transwell chemotaxis and coculture assays of mouse splenic CD8^+^ T cells (created with Figdraw). (B) Transwell migration results of splenic CD8^+^ T cells cocultured with HCC cells transduced with shPGK1 or shNC (*n* = 3). (C) Flow cytometry analysis of M2‐type TAMs infiltration in shPGK1 and shNC Hep53.4 orthotopic HCC models (*n* = 5). (D) Representative immunofluorescence images and comparison of the number of positive cells for CD8 and CD68 in PGK1 high expression (*n* = 37) and PGK1 low expression samples (*n* = 37) from HCC patients. (E) Schematic illustration of Hep53.4 or Hepa1‐6 orthotopic HCC models in C57BL/6J mice, along with intraperitoneal injection of anti‐CSF1R antibody. (F) Gross images, tumor volume, and tumor weight of shPGK1 and shNC Hep53.4 orthotopic tumors, with or without anti‐CSF1R antibody treatment (*n* = 5). (G) M2‐type TAMs infiltration (CD206^+^ TAMs) in shPGK1 and shNC Hep53.4 orthotopic tumors with or without anti‐CSF1R antibody treatment. (H) Comparison of CD8^+^ T cells infiltration in shPGK1 and shNC Hep53.4 orthotopic tumors with or without anti‐CSF1R antibody treatment (*n* = 5). (I) Comparison of Granzyme B^+^, perforin^+^, IFN‐γ^+^, and TNF‐α^+^ CD8^+^ T cells in Hep53.4 orthotopic HCC models with or without anti‐CSF1R antibody treatment (*n* = 5). (J) Schematic illustration of mouse BMDMs transwell coculture assay (created with Figdraw). (K) The expression of macrophage M2 marker (CD206) and M1 marker (iNOS) of BMDMs cocultured with shPGK1 or shNC Hep53.4 and Hepa1‐6 cells by flow cytometry (*n* = 3). (L) Transwell results for BMDMs cocultured with shPGK1 or shNC Hep53.4 and Hepa1‐6 cells (*n* = 3). (M) Comparison of CFSE‐labeled mouse splenic CD8^+^ T cells proliferation after coculture with differently treated BMDMs pre‐cocultured with Hep53.4 cells (*n* = 3). (N) Effector molecules expression (Granzyme B, perforin, IFN‐γ, and TNF‐α) in CD8^+^ T cells cocultured with differently treated BMDMs pre‐cocultured with Hep53.4 cells (*n* = 3). TAMs, tumor‐associated macrophages. BMDMs, bone marrow‐derived macrophages. Data were presented as mean ± SEM. ns, no significant difference. ^*^ *p* < 0.05; ^**^ *p* < 0.01; ^***^ *p* < 0.001; ^****^ *p* < 0.0001.

We next sought to explore potential mechanisms by which PGK1 inhibits CD8^+^ T cells. In vitro transwell chemotaxis assay and coculture assay were used to examine the impact of PGK1 on CD8^+^ T cells (Figure 3A). The chemotaxis assay showed shPGK1 and shNC HCC cells exhibited similar ability to attract CD8^+^ T cells (Figure 3B). The proliferation and cytotoxic activity of CD8^+^ T cells cocultured with PGK1 knockdown HCC cells were comparable to those in the control group (Figure S3A,B). These results indicated tumor‐derived PGK1 had no direct effect on CD8^+^ T cells. Therefore, we speculated that PGK1 might indirectly regulate CD8^+^ T cells through other mechanisms. TAMs, which are considered to have an M2 immunosuppressive phenotype, are the primary immune cell populations in the HCC TME. We and others have previously demonstrated that TAMs inhibit the infiltration of CD8^+^ T cells into the TME and suppress their activity within the tumor [25, 26, 27, 28]. Analyses of the TCGA database and flow cytometry of clinical HCC samples both demonstrated a positive correlation between PGK1 expression and M2‐type TAMs infiltration (Figure S3C,D). A substantially decreased proportion of M2‐type TAMs was also observed after PGK1 knockdown in the orthotopic tumor models in C57BL/6J mice (Figure 3C and Figure S3E). Moreover, the immunofluorescence assay revealed that human HCC samples with high PGK1 expression exhibited extensive macrophage infiltration accompanied by relatively low CD8^+^ T cell abundance (Figure 3D). We therefore hypothesized that PGK1 may exert an immunosuppressive effect on CD8^+^ T cells through TAMs. Because CSF1R signaling is critical for the survival and maintenance of TAMs, particularly M2‐type immunosuppressive TAMs, targeting CSF1R represents a complementary strategy to preferentially deplete or reprogram this population in the TME [29, 30, 31]. Accordingly, we next employed an in vivo anti‐CSF1R antibody to suppress M2‐type TAMs in the orthotopic tumor models (Figure 3E). As expected, depletion of M2‐type TAMs by CSF1R blockade significantly inhibited the growth of orthotopic liver tumors (Figure 3F,G and Figure S3F,G), accompanied by increased infiltration and enhanced functionality of CD8^+^ T cells (Figure 3H,I, Figure S3H,I). Moreover, the difference between the shPGK1 and shNC groups was nearly completely abrogated following inhibition of M2‐type TAMs. To identify the effect of tumor cell‐intrinsic PGK1 on macrophages, bone marrow‐derived macrophages (BMDMs) were incubated in a noncontact coculture system to mimic the microenvironment interactions (Figure 3J). Flow cytometric analysis showed that BMDMs cocultured with shPGK1 HCC cells had reduced M2 polarization and enhanced M1 polarization (Figure 3K). Consistent results were obtained in macrophages differentiated from THP‐1 cells (Figure S3J). Furthermore, BMDMs and THP‐1 cocultured with shPGK1 HCC cells displayed markedly attenuated migratory ability compared to those cocultured with shNC cells (Figure 3L and Figure S3K). To further delineate the role of M2‐type macrophages in PGK1‐mediated suppression of CD8^+^ T cells, BMDMs were cocultured with CD8^+^ T cells. BMDMs preconditioned with shPGK1 HCC cells significantly enhanced the proliferation and effector function of CD8^+^ T cells compared with the shNC group. In contrast, IL‐4–induced M2 polarization of BMDMs markedly impaired CD8^+^ T cell responses, abolishing the differences between the shPGK1 and shNC groups (Figure 3M,N and Figure S3L,M). Taken together, these findings suggested that HCC‐derived PGK1 promotes TAMs recruitment and M2 polarization, thereby suppressing the infiltration and function of CD8^+^ T cells.

### 2.4 PGK1 Enhances the Recruitment and M2 Polarization of TAMs in a CCL2‐dependent Manner

**FIGURE 4:**
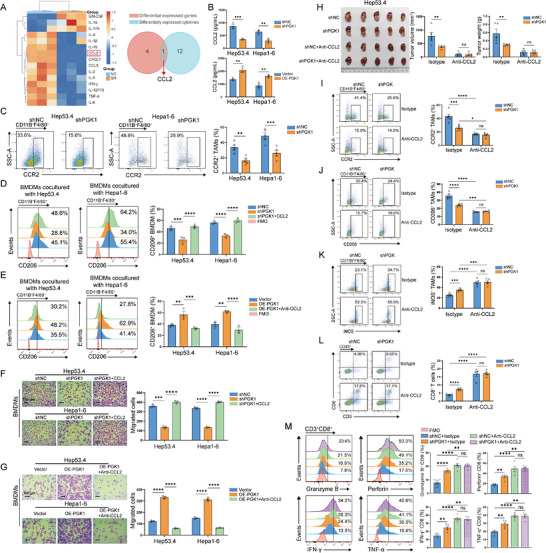
PGK1 enhances the recruitment and M2 polarization of TAMs in a CCL2‐dependent manner. (A) Heatmap showing the relative cytokine expression in multiplex secretome analysis of supernatants from Hep53.4 cells transduced with shPGK1 or shNC (left), and Venn diagram of the overlap between differentially expressed cytokine genes from transcriptome sequencing and differentially expressed cytokines identified by multiplex secretome analysis (right). (B) ELISA quantification of CCL2 level in cell supernatants of Hep53.4 and Hepa1‐6 (*n* = 3). (C) Infiltration of CCR2^+^ TAMs in shPGK1 or shNC Hep53.4 and Hepa1‐6 orthotopic HCC models (*n* = 5). (D) Expression of the M2‐type macrophage marker CD206 in BMDMs cocultured with the indicated HCC cells, with or without supplementation of recombinant CCL2 protein (100 ng/mL) (*n* = 3). (E) Expression of the M2‐type macrophage marker CD206 in BMDMs cocultured with the indicated HCC cells, with or without supplementation of anti‐CCL2 antibody (5 µg/mL) (*n* = 3). (F) Transwell results for BMDMs cocultured with the indicated HCC cells, with or without supplementation of recombinant CCL2 protein (100 ng/mL) (*n* = 3). (G) Transwell results for BMDMs cocultured with the indicated HCC cells, with or without supplementation of anti‐CCL2 antibody (5 µg/mL) (*n* = 3). (H) Gross images, tumor volume, and tumor weight of shPGK1 and shNC Hep53.4 orthotopic tumors in C57BL/6J mice, with or without anti‐CCL2 antibody treatment (*n* = 5). (I) Flow cytometry analysis of CCR2^+^ TAMs infiltration in Hep53.4 orthotopic HCC models with or without anti‐CCL2 antibody treatment (*n* = 5). (J) Comparison of M2‐type TAMs Infiltration in Hep53.4 orthotopic HCC models with or without anti‐CCL2 antibody treatment (*n* = 5). (K) Comparison of M1‐type TAMs (iNOS^+^ TAMs) infiltration in Hep53.4 orthotopic HCC models with or without anti‐CCL2 antibody treatment (*n* = 5). (L) Comparison of CD8^+^ T cell infiltration in Hep53.4 orthotopic HCC models with or without anti‐CCL2 antibody treatment (*n* = 5). (M) Comparison of Granzyme B^+^, perforin^+^, IFN‐γ^+^, and TNF‐α^+^ CD8^+^ T cells in Hep53.4 orthotopic HCC models with or without anti‐CCL2 antibody treatment (*n* = 5). Data were presented as mean ± SEM. ns, no significant difference. ^*^ *p* < 0.05; ^**^ *p* < 0.01; ^***^ *p* < 0.001; ^****^ *p* < 0.0001.

Next, we further investigated the mechanisms underlying PGK1‐mediated TAMs recruitment and M2 polarization. Multiplex secretome analysis of Hep53.4 cells transduced with shPGK1 or shNC revealed that, among the significantly altered cytokines, only CCL2 was concurrently downregulated in the transcriptome sequencing (Figure 4A and Figure S4A). ELISA and Western blot further confirmed this finding (Figure 4B and Figure S4B). CCL2 is a well‐known key mediator that promotes macrophage chemotaxis and polarization through the combination with C‐C motif chemokine receptor 2 (CCR2) [32]. We next sought to investigate whether CCL2 mediates the effects of tumor‐intrinsic PGK1 on macrophage regulation. In tumor samples from HCC patients, we found that CCR2^+^ TAMs were significantly more abundant in the PGK1 high expression group compared to the low expression group (Figure S4C). In orthotopic HCC models in mice, the infiltration of CCR2^+^ TAMs was markedly reduced in the shPGK1 group (Figure 4C). Coculture experiments showed that recombinant CCL2 supplementation rescued the decrease in BMDM M2 polarization caused by PGK1 knockdown, whereas anti‐CCL2 antibody treatment abrogated the increase in M2 polarization induced by PGK1 overexpression (Figure 4D,E). Similar results were observed in the THP‐1–HCC cells coculture system (Figure S4D,E). Consistently, chemotaxis assays demonstrated that recombinant CCL2 supplementation or anti‐CCL2 antibody treatment reversed the alterations in macrophage chemotaxis induced by PGK1 knockdown or overexpression, respectively (Figure 4F,G and Figure S4F,G). In vivo experiments demonstrated that treatment with an anti‐CCL2 antibody significantly diminished tumor burden and reduced the proportions of CCR2^+^ TAMs and M2‐type TAMs in the orthotopic HCC models, while increasing the infiltration of M1‐type TAMs (Figure 4H–K and Figure S4H–K). Furthermore, anti‐CCL2 treatment resulted in comparable tumor size, tumor weight, TAMs composition, and the infiltration and functional activity of CD8^+^ T cells between shNC and shPGK1 tumors (Figure 4L,M and Figure S4L,M). These findings indicated that tumor‐intrinsic PGK1 drives CCL2 secretion, thereby modulating TAMs recruitment and M2 polarization, which in turn impairs the infiltration and effector activity of CD8^+^ T cells.

### 2.5 PGK1 Upregulates CCL2 Expression by Activating AKT/GSK‐3β/β‐Catenin Signaling Pathway in HCC Cells

**FIGURE 5:**
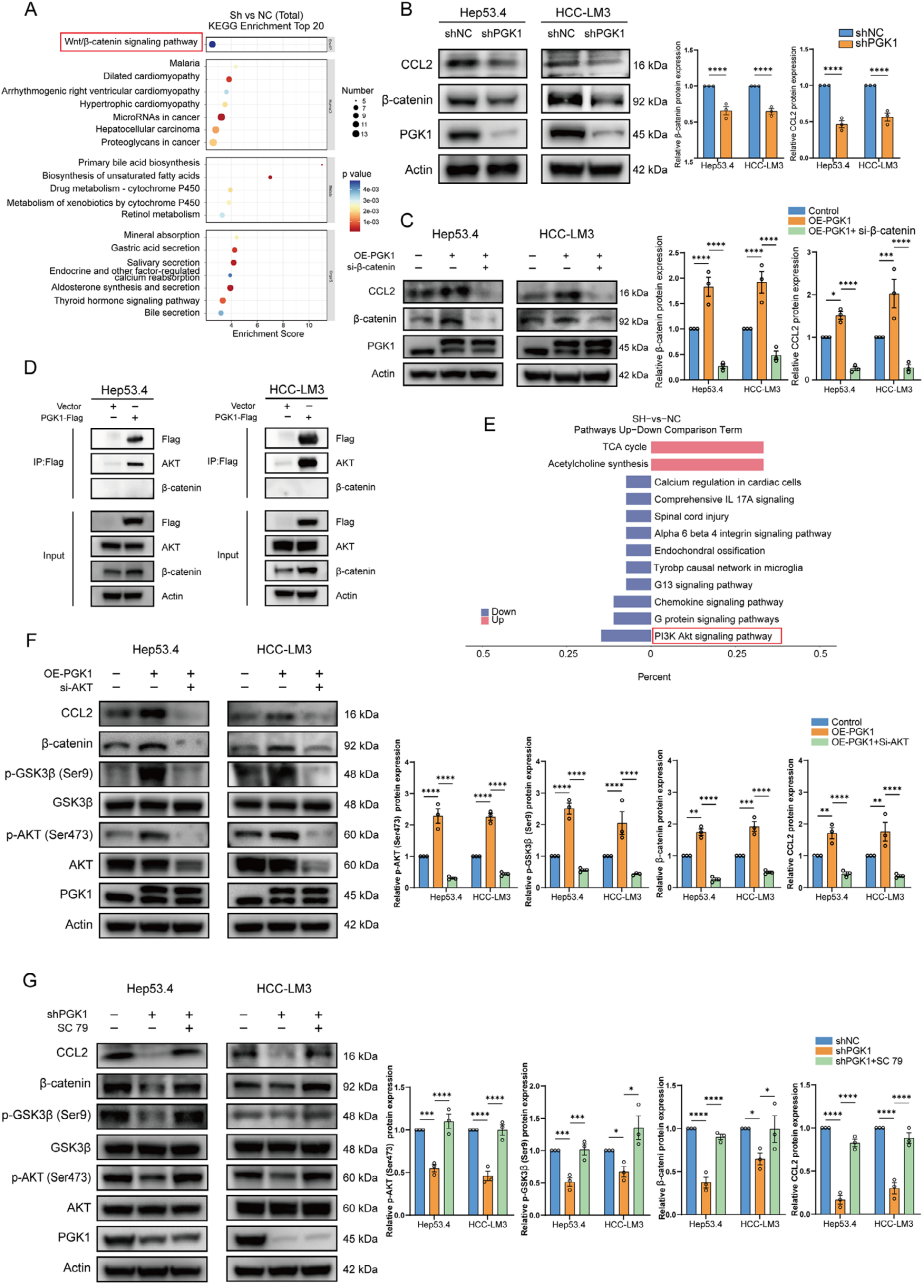
PGK1 upregulates CCL2 expression by activating AKT/GSK‐3β/β‐catenin signaling pathway in HCC cells. (A) Top 20 significantly enriched KEGG pathways in transcriptome sequencing of Hep53.4 cells transduced with shPGK1 or shNC. (B) Western blot analysis of β‐catenin and CCL2 expression in PGK1 knockdown and control HCC cells (*n* = 3). (C) Western blot analysis of CCL2 expression in PGK1‐overexpressing HCC cells with or without pre‐established β‐catenin knockdown (*n* = 3). (D) Co‐IP assays showing whether β‐catenin and AKT interact with PGK1 in HCC cells. (E) Significantly enriched pathway terms identified in the phosphoproteomics analysis. (F) Western blot analysis of p‐AKT (Ser473), p‐GSK3β (Ser9), β‐catenin, and CCL2 expression in PGK1‐overexpressing HCC cells with or without pre‐established AKT knockdown (*n* = 3). (G) Western blot analysis of p‐AKT (Ser473), p‐GSK3β (Ser9), β‐catenin, and CCL2 in PGK1 knockdown HCC cells treated with or without the AKT activator SC79 (10 µm) (*n* = 3). Data were presented as mean ± SEM. ns, no significant difference. ^*^ *p* < 0.05; ^**^ *p* < 0.01; ^***^ *p* < 0.001; ^****^ *p* < 0.0001.

We next sought to elucidate the molecular mechanisms by which tumor cell–intrinsic PGK1 regulates CCL2 expression in HCC. Transcriptomic sequencing results revealed that the Wnt/β‐catenin pathway was significantly enriched according to KEGG analysis (Figure 5A). Notably, β‐catenin has been identified as a key transcriptional  regulator driving CCL2 transcription [33]. Western blot results demonstrated that PGK1 knockdown markedly reduced the protein expression of β‐catenin and CCL2, while PGK1 overexpression significantly upregulated β‐catenin and CCL2 (Figure 5B,C and Figure S5A,B). Importantly, PGK1 overexpression–induced upregulation of CCL2 was abolished by prior knockdown of β‐catenin (Figure 5C and Figure S5B). These results indicated that β‐catenin mediates the regulatory effect of PGK1 on CCL2. However, RT‐PCR analysis revealed that PGK1 knockdown did not affect β‐catenin mRNA levels, indicating no direct transcriptional regulation (Figure S5C). Given that PGK1 is a protein kinase, we investigated whether it directly binds to β‐catenin. However, Co‐IP showed that PGK1 does not interact with β‐catenin, suggesting that it regulates β‐catenin protein expression indirectly through alternative pathways (Figure 5D and Figure S5D). Phosphoproteomic analysis revealed that PI3K/AKT signaling was markedly downregulated following PGK1 knockdown (Figure 5E), consistent with previous reports that PGK1 could activate the AKT pathway [34, 35]. Given that AKT is a critical upstream regulator of β‐catenin, promoting cytoplasmic stabilization and nuclear translocation of β‐catenin by phosphorylating GSK3β [36]. Therefore, we further hypothesized that PGK1 may regulate β‐catenin protein expression through AKT activation, thereby promoting CCL2 transcription. Experimental results confirmed that PGK1 is able to directly bind to AKT, thereby promoting its phosphorylation and the activation of downstream signaling pathways (Figure 5D and Figure S5D). Furthermore, Prior AKT knockdown suppressed the upregulation of β‐catenin and CCL2 induced by PGK1 overexpression (Figure 5F and Figure S5E). Correspondingly, the AKT activator SC79 rescued the downregulation of β‐catenin and CCL2 caused by PGK1 knockdown (Figure 5G and Figure S5F). Taken together, these findings demonstrated that HCC–derived PGK1 promotes CCL2 expression via activation of the AKT/GSK3β/β‐catenin pathway.

### 2.6 Pharmacological Targeting of PGK1 Improves the Response of HCC to Anti‐PD‐L1 Therapy

**FIGURE 6:**
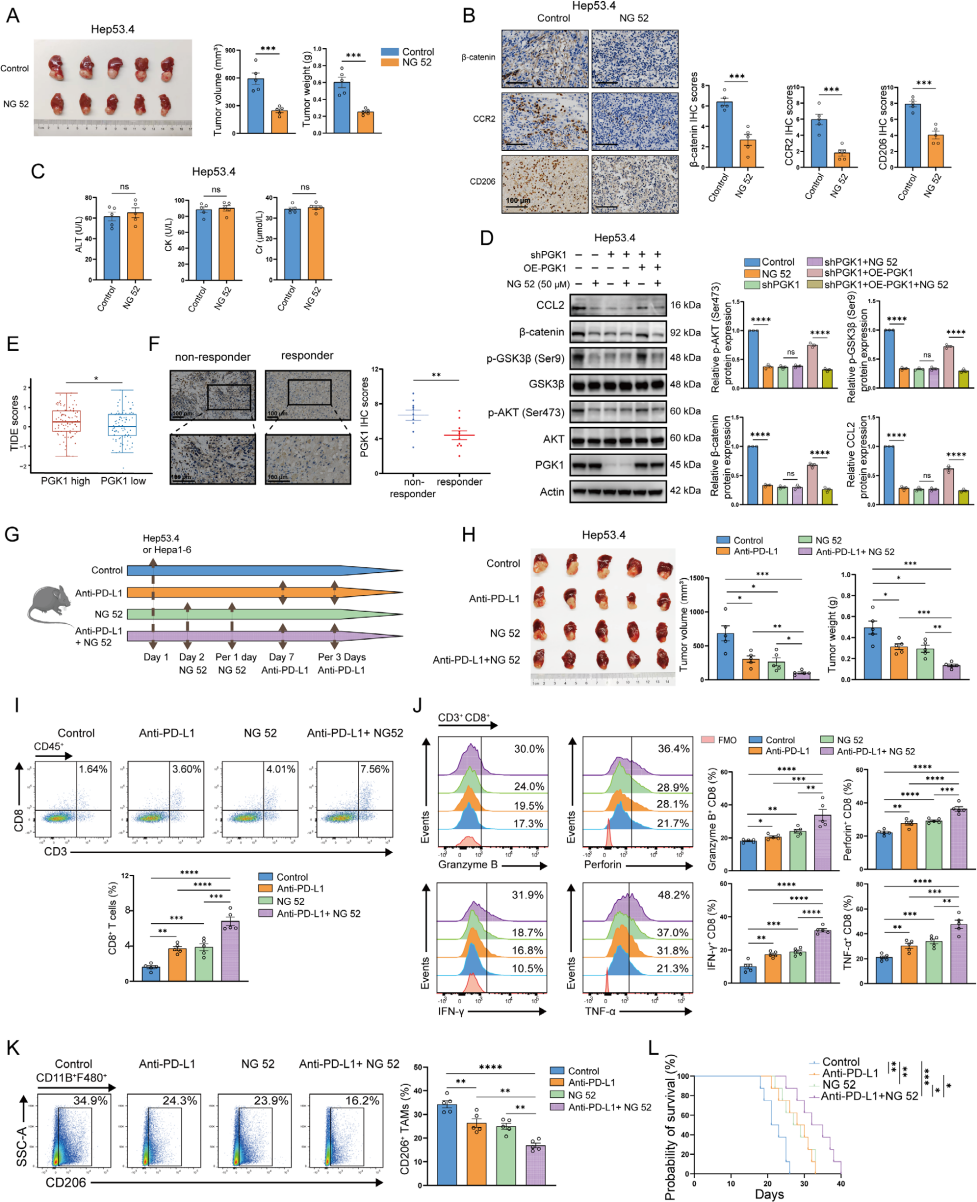
Pharmacological targeting of PGK1 improves the response of HCC to anti‐PD‐L1 therapy. (A) Gross images, tumor volume, and tumor weight of Hep53.4 orthotopic HCC models in C57BL/6J mice, with or without PGK1 inhibitor NG 52 treatment (*n* = 5). (B) Representative IHC staining images (400x magnification) and comparison of IHC scores for β‐catenin, CCR2, and CD206 in Hep53.4 orthotopic tumors, with or without NG 52 treatment (*n* = 5). (C) Serum ALT, CK, and Cr levels in mice with Hep53.4 orthotopic tumors, with or without NG 52 treatment (*n* = 5). (D) Western blot analysis of p‐AKT (Ser473), p‐GSK3β (Ser9), β‐catenin, and CCL2 in Hep53.4 cells under different treatments (*n* = 3). (E) Comparison of TIDE prediction scores between HCC samples with high and low PGK1 expression in the TCGA database. (F) Representative IHC images (200x and 400x magnification) and comparison of IHC scores for PGK1 in HCC patients with or without response to immunotherapy, representing the responder (*n* = 10) and non‐responder groups (*n* = 10). (G) Schematic of Hep53.4 or Hepa1‐6 orthotopic HCC models treated with anti–PD‐L1 antibody, NG 52, or the combination. (H) Gross images, tumor volume, and tumor weight of Hep53.4 orthotopic tumors treated with anti–PD‐L1 antibody, NG 52, or the combination (*n* = 5). (I) Flow cytometry analysis of CD8^+^ T cell infiltration in Hep53.4 orthotopic HCC models subjected to different treatments (*n* = 5). (J) Comparison of Granzyme B^+^, perforin^+^, IFN‐γ^+^, and TNF‐α^+^ CD8^+^ T cells in Hep53.4 orthotopic HCC models subjected to different treatments (*n* = 5). (K) Infiltration of M2‐type TAMs in Hep53.4 orthotopic HCC models with different treatments (*n* = 5). (L) Survival of Hep53.4 orthotopic HCC models with various treatments (*n* = 8). HR and 95% CI: Anti–PD‐L1 vs. Control, 0.1481 (0.0386–0.5673); NG 52 vs. Control, 0.1685 (0.0456–0.6219); Combination vs. Control, 0.0616 (0.0141–0.2703); Combination vs. Anti–PD‐L1, 0.2736 (0.0811–0.9238); Combination vs. NG 52, 0.2883 (0.0871–0.9543). ALT, alanine aminotransferase. Cr, creatinine. CK, creatine kinase. Data were presented as mean ± SEM. ns, no significant difference. ^*^ *p* < 0.05; ^**^ *p* < 0.01; ^***^ *p* < 0.001; ^****^ *p* < 0.0001.

Next, we explored the therapeutic potential of PGK1 targeting in HCC. NG52, a selective PGK1 inhibitor previously reported to inhibit glioblastoma [37], significantly suppressed tumor progression in the orthotopic HCC models (Figure 6A and Figure S6A). Consistently, IHC staining demonstrated that NG 52 treatment markedly reduced the expression of β‐catenin, CCR2, and CD206 in tumor tissues, supporting inhibition of the PGK1‐driven signaling axis and its downstream immunomodulatory effects (Figure 6B and Figure S6B). Moreover, serum biochemical analyses showed no significant differences in alanine aminotransferase (ALT), creatinine (Cr), and creatine kinase (CK) levels between the treatment and control groups, indicating that the inhibitor did not induce detectable systemic toxicity and exhibited a favorable safety profile (Figure 6C and Figure S6C). Western blot analysis further demonstrated that NG 52 significantly suppressed the AKT/GSKβ/β‐catenin signaling pathway and downstream CCL2 expression. Notably, this inhibitory effect was abolished following PGK1 knockdown, whereas re‐expression of PGK1 in shPGK1 HCC cells restored the response to NG 52, suggesting that the effects of NG 52 are predominantly mediated by on‐target inhibition of PGK1 (Figure 6D and Figure S6D). Using the TIDE algorithm, we predicted the immunotherapy response of TCGA HCC patients with different PGK1 expression levels [38]. The analysis suggested that patients with low PGK1 expression may derive greater benefit from immunotherapy, suggesting a potential role for PGK1 as a predictive biomarker (Figure 6E). For validation, we analyzed an independent clinical cohort of HCC patients receiving anti‐PD‐1/PD‐L1 therapy. Patients exhibiting partial response (PR) or progressive disease (PD), as determined according to RECIST criteria, were classified as responder and non‐responder groups, respectively. Corresponding tumor samples and clinical data were collected (Table S2). IHC analysis indicated that tumor tissues from responders exhibited significantly lower PGK1 expression than those from non‐responders (Figure 6F). These findings provided preliminary clinical evidence supporting an association between low PGK1 expression and improved response to immunotherapy.

Having revealed that PGK1 exerts its effects on CD8^+^ T cells by promoting TAMs recruitment and M2 polarization, we then investigated whether pharmacological inhibition of PGK1 using NG 52 could enhance the therapeutic efficacy of immunotherapy in an orthotopic HCC model (Figure 6G). We observed that either NG 52 or anti‐PD‐L1 antibody alone showed some degree of tumor growth suppression compared with the control group (Figure 6H and Figure S6E). Notably, combined therapy exhibited significantly greater antitumor efficacy in vivo than either monotherapy. Flow cytometry analysis revealed that the combination treatment significantly enhanced both the infiltration and cytotoxic capacity of CD8^+^ T cells, while markedly reducing the infiltration of M2‐type TAMs (Figure 6I–K and Figure S6F–H). Moreover, combined therapy of NG 52 and anti‐PD‐L1 antibody significantly prolonged the survival of tumor‐bearing mice (Figure 6L and Figure S6I). These results demonstrated that pharmacological targeting of PGK1 could substantially improve the efficacy of anti‐PD‐L1 therapy in HCC, suggesting its potential as a novel therapeutic target for combinatorial immunotherapy.

## 3 Discussion

Advanced HCC remains an aggressive tumor with a poor prognosis characterized by a highly suppressive immune microenvironment [39]. TAMs, typically exhibiting an M2 protumor phenotype, play a crucial role in tumor immune escape and progression [34, 40]. They are capable of suppressing either directly or indirectly the recruitment and activation of CD8^+^ T cells [13, 41]. Therefore, given their role as central mediators of immune suppression in the TME, therapeutic strategies targeting TAMs, such as altering their phenotype or frequency and targeting related regulators, may provide new directions and breakthroughs for antitumor therapy of HCC [42, 43].

Currently, the research on the association between PGK1 and tumor immunity, particularly in HCC, remains a critical gap that needs to be addressed. Therefore, elucidating the specific role of PGK1 in regulating tumor immune responses is an indispensable aspect of future investigations. Our study highlights the importance of tumor cell‐intrinsic PGK1 in shaping the immune landscape of HCC, demonstrating that its knockdown reduces M2‐type TAMs proportion while promoting the infiltration and function of CD8^+^ T cells. In addition, in tumor treatment experiments, results indicated that pharmacological inhibition of PGK1 enhances the efficacy of the anti‐PD‐L1 antibody, suggesting a synergistic interaction between the two treatments.

In addition to its well‐established role in regulating glucose metabolism, the protein kinase function of PGK1 has also attracted great attention in recent years. It was previously reported that PGK1 induces autophagy by phosphorylating Beclin at S30 in tumorigenesis under glutamine deprivation or hypoxia [19]. Another study showed PGK1 directly phosphorylates PRAS40 to promote HCC cell proliferation through repressing autophagy‐mediated cell death under normoxia [44]. Similarly, in our study, we found PGK1 can interact with AKT, thereby enhancing its phosphorylation and activating downstream signaling pathways. This leads to the accumulation and increased expression of cytosolic β‐catenin, consequently promoting CCL2 transcription and expression, ultimately fostering an HCC microenvironment characterized by high infiltration of immunosuppressive TAMs. Previous studies have primarily focused on the role of PGK1 in promoting HCC cell proliferation and tumorigenesis, while our study highlights the regulatory role of PGK1 in the immune microenvironment of HCC. We believe these findings are not contradictory and instead suggest the functional diversity of PGK1 in tumor progression. Owing to its instrumental role in the occurrence and development of multiple malignant tumors, PGK1 is an attractive therapeutic target for cancer therapy. Inhibitors targeting the PGK1 exhibited potent antitumor activity in vitro and in vivo [45, 46]. In this study, we found that targeting PGK1 with NG 52, an effective and selective kinase inhibitor, exerted a potent antitumor effect by modulating the tumor immune microenvironment in orthotopic HCC models. Nevertheless, this finding still requires validation in additional tumor models. In addition, because PGK1 is broadly expressed across diverse cell types and fulfills multiple functional roles, the development of tumor‐specific targeting strategies is essential. Emerging nanoparticle‐based and biomaterial‐assisted delivery platforms provide a promising strategy for achieving localized retention and microenvironment‐specific targeting of therapeutic agents, offering a rational framework for integrating targeted delivery into PGK1‐directed therapeutic design [47, 48]. Building on this concept, inhibition of tumor cell–intrinsic PGK1 could be incorporated into combination tumor immunotherapy by selectively reprogramming TME, thereby alleviating immunosuppressive signaling and potentially enhancing the efficacy of ICIs.

The crosstalk among different types of cells in the TME has been widely studied and plays an important role in tumor progression. Cytokines have recently been identified as mediators of crosstalk in the communication between tumor cells and macrophages. Previous studies have demonstrated that CCL2 is a highly expressed chemokine in tumor cells and can promote the recruitment of TAMs and their polarization to the M2 phenotype through interaction with its receptor, CCR2. Blockade of the CCL2/CCR2 axis can markedly reverse the immunosuppressive microenvironment and elicit potent antitumor CD8^+^ T cell responses [32, 49]. Our study indicated that CCL2 production in HCC cells is partially mediated by PGK1‐activated AKT/GSK‐3β/β‐catenin signaling. Furthermore, the PGK1‐driven immunosuppressive microenvironment in HCC depends on CCL2. These findings suggested that CCL2 is a critical mediator in PGK1‐induced recruitment and M2‐type polarization of TAMs, which subsequently suppresses the antitumor efficacy of CD8^+^ T cells. Accordingly, targeting the CCL2/CCR2 signaling may serve as a promising therapeutic strategy for HCC immunotherapy.

Recent advances in single‐cell RNA sequencing (scRNA‐seq) and spatial transcriptomics have revealed substantial TAMs diversity beyond the M1/M2 framework and underscored the importance of immune cell organization in shaping antitumor responses [50, 51]. The interplay between TAMs and CD8^+^ T cells forms a critical axis that shapes the TME and influences the efficacy of cancer immunotherapy. Notably, TAMs have been reported to localize in close spatial proximity to CD8^+^ T cells and directly interact with them, thereby driving CD8^+^ T cells dysfunction and exhaustion through diverse mechanisms, including metabolic regulation and cytokine‐mediated signaling [52]. A study identified seven transcriptionally distinct TAM subtypes by scRNA‐seq, and spatial transcriptomic analysis showed that the gene signatures of all subtypes were closely associated with T cell exhaustion programs, indicating that TAMs heterogeneity may serve as a key upstream regulator of CD8^+^ T cell functional states [53]. Integrated multi‐modal analyses, including scRNA‐seq, spatial transcriptomics, and multiplex immunofluorescence, identified an SPP1^+^/TREM2^+^ TAM subpopulation enriched at metastatic sites in prostate cancer, and blockade of SPP1 signaling remodeled the TME and enhanced CD8^+^ T cell infiltration [54]. Our study relied on multiparameter flow cytometry, which lacks spatial resolution and is inherently limited in capturing the full heterogeneity of the macrophage compartment. Therefore, future studies integrating single‐cell and spatially resolved approaches will be essential to further elucidate the crosstalk between PGK1 in HCC cells and TAMs or CD8^+^ T cells, and to improve immunotherapeutic strategies.

In conclusion, we uncovered a novel mechanistic link between high PGK1 expression in tumor cells, increased CCL2 secretion, enrichment of M2‐type TAMs, and the subsequent reduction in CD8^+^ T cell infiltration and activation, collectively contributing to an immunosuppressive TME in HCC. These findings highlighted the potential of PGK1 as a therapeutic target for combination immunotherapy. Overall, our study established a critical role for PGK1 in shaping the immune landscape of HCC at the molecular level and provided a foundation for future research that may extend to other tumor types, thereby deepening our understanding of PGK1 in tumor immunobiology.

## 4 Methods

### 4.1 Cell Culture and Transfection

The detailed methods are described and provided in the Supporting Information.

### 4.2 Mouse Tumor Models and Treatment

Experimental mice were raised in a specific pathogen‐free (SPF) environment. All animal experiments were undertaken with review and approval from the animal Ethics Committee of the First Affiliated Hospital, Zhejiang University School of Medicine (Approval number: 2025‐134)and complied with the National Research Council's Guide for the Care and Use of Laboratory Animals. The establishment and treatment of mouse tumor models are mentioned in detail in the Supporting Information.

### 4.3 Single‐Cell Suspension Preparation and Flow Cytometry

Mice were euthanized at the end of each experimental time point, and tumor tissues were harvested, digested, and prepared into a single‐cell suspension using a 70‐µm cell strainer. Relevant details are provided in the Supporting Information. Then, single‐cell suspensions were incubated with fluorochrome‐conjugated antibodies according to the manufacturer's instructions. Lymphocytes were first gated on forward scatter (FSC) and side scatter (SSC) properties, then doublets and dead cells were excluded. Immune cells were then selected as CD45^+^ cells for subsequent subset analysis. CD4^+^ T cells were identified as CD45^+^CD3^+^CD4^+^ cells, and CD8^+^ T cells were identified as CD45^+^CD3^+^CD8^+^ cells. NK cells were defined as CD45^+^CD3^−^NK1.1^+^ cells, whereas B cells were defined as CD45^+^CD3^−^CD19^+^ cells. Dendritic cells (DCs) were identified as CD45^+^F4/80^−^CD11c^+^ cells. Regulatory T cells (Tregs) were identified as CD45^+^ CD3^+^CD4^+^CD25^+^FOXP3^+^ cells. Macrophages subsets were characterized as M2 macrophages (CD45^+^CD11b^+^F4/80^+^CD206^+^ or CD45^+^CD11b^+^CD68^+^CD206^+^), M1 macrophages (CD45^+^CD11b^+^F4/80^+^iNOS^+^ or CD45^+^CD11b^+^CD68^+^CD86^+^), and CCR2^+^ macrophages (CD45^+^CD11b^+^F4/80^+^CCR2^+^ or CD45^+^CD11b^+^CD68^+^CCR2^+^). For intracellular staining, cells were fixed and permeabilized before staining with the indicated antibodies. The effector function of CD8^+^ T cells was evaluated based on intracellular expression levels of TNF, granzyme B, IFN‐γ, and perforin in CD45^+^CD3^+^CD8^+^ T cells. Data were acquired on a flow cytometer and analyzed using FlowJo 10.

The following fluorochrome‐conjugated antibodies from BioLegend (USA) were used for flow cytometric analysis: Zombie NIR Fixable Viability Kit (423106), Zombie Aqua Fixable Viability Kit, PE/Cyanine7 anti‐mouse CD45 Antibody (103114), FITC anti‐mouse CD45 Antibody (103108), Brilliant Violet 605 anti‐mouse CD45 (103155), Antibody PerCP/Cyanine5.5 anti‐mouse CD8a Antibody (100734), APC anti‐mouse CD3 Antibody (100236), PerCP/Cyanine5.5 anti‐mouse CD3 Antibody (100218), PE anti‐mouse CD4 Antibody (100408), PE/Cyanine7 anti‐mouse CD4 Antibody (100528), FITC anti‐mouse CD19 Antibody (115505), PE anti‐mouse NK‐1.1 Antibody (108708), APC anti‐mouse CD25 Antibody (102012), PE anti‐mouse FOXP3 Antibody (126404), FITC anti‐mouse/human CD11b Antibody (101206), PerCP/Cyanine5.5 anti‐mouse F4/80 Antibody (123128), APC anti‐mouse CD11c (117310), Antibody APC anti‐mouse CD206 (MMR) Antibody (141708), PE anti‐Nos2 (iNOS) Antibody (696806), Brilliant Violet 785 anti‐mouse CD192 (CCR2) Antibody (150621), Alexa Fluor 647 anti‐human/mouse Granzyme B Antibody (515406), PE anti‐mouse Perforin Antibody (154306), Brilliant Violet 711 anti‐mouse TNF‐α Antibody (506349), Brilliant Violet 605 anti‐mouse IFN‐γ Antibody (505839), Brilliant Violet 421 anti‐human CD45 Antibody (304032), PE/Cyanine7 anti‐human CD3 Antibody (300420), FITC anti‐human CD8 Antibody (344704), APC/Cyanine7 anti‐mouse/human CD11b Antibody (101226), PerCP/Cyanine5.5 anti‐human CD68 Antibody (333813), APC anti‐human CD206 (MMR) Antibody (321110), Brilliant Violet 650 anti‐human CD86 Antibody (305428), PE anti‐human CD192 (CCR2) Antibody (357206).

### 4.4 Human Samples

A total of 74 HCC specimens and 52 noncancerous liver tissue samples were collected from patients with primary HCC who underwent curative resection between 2016 and 2019 at the First Affiliated Hospital, Zhejiang University School of Medicine. Tumor samples were collected from 20 patients with HCC who received anti–PD‐1/PD‐L1 therapy at Shulan (Hangzhou) Hospital between 2019 and 2022. These samples were used for Western blot, immunohistochemistry, immunofluorescence, and survival analysis. In addition, fresh tumor surgical samples from 16 HCC patients were collected for quantitative RT‐PCR and flow cytometry analysis. The study was approved by the Ethics Committee of the First Affiliated Hospital, Zhejiang University School of Medicine (Approval number: IIT2025B1047) and the Ethics Committee of the Shulan (Hangzhou) Hospital (Approval number: KY2025114). Written informed consent was obtained from patients before tissue collection for this study. The study was conducted in accordance with the principles outlined in the Declaration of Helsinki.

### 4.5 Immunohistochemistry and Immunofluorescence

Tissue sections of 3 µm thickness were mounted on glass slides for staining. Five areas from each section were randomly selected and assessed. Detailed protocols of immunohistochemistry (IHC) and immunofluorescence are provided in the Supporting Information.

### 4.6 Western Blot and Co‐Immunoprecipitation

Experimental details of Western blot and Co‐immunoprecipitation (Co‐IP) are given in the Supporting Information.

### 4.7 RNA Extraction and Quantitative RT‐PCR

The detailed steps of RNA extraction and quantitative RT‐PCR are shown in the Supporting Information.

### 4.8 Transcriptomic Sequence and Phosphoproteomic Analysis

Transcriptomic sequence and phosphoproteomic analysis were performed with shPGK1 and shNC Hep53.4 cells (*n* =  3) by OE Biotech Co., Ltd. (Shanghai, China).

For transcriptomic sequence, the libraries were sequenced on an Illumina Novaseq 6000 platform, and 150 bp paired‐end reads were generated. Raw reads of fastq format were first processed to obtain the clean reads. Then, about 6.88–7.06 G clean reads for each sample were retained for subsequent analyses. The clean reads were mapped to the reference genome using HISAT2. FPKM of each gene was calculated, and the read counts of each gene were obtained by HTSeq‐count. In the differential expression analysis, an adjusted *p*‐value < 0.05 and |FoldChange|>1.5 were set as the threshold for significant differential expression. Bioinformatic analysis was performed using the OECloud tools at https://cloud.oebiotech.com/task/. For phosphoproteomic analysis, Tandem mass tag (TMT) labeling was used to label different samples. Total protein was extracted from the samples, with a portion reserved for protein concentration determination and SDS‐PAGE analysis. Another aliquot was subjected to tryptic digestion and labeling. Subsequently, equal amounts of each labeled sample were pooled for phosphopeptide enrichment. Finally, the samples were analyzed by LC‐MS/MS, followed by comprehensive data analysis. OECloud tools (https://cloud.oebiotech.com/task/) were used to conduct all bioinformatics analyses.

### 4.9 TCGA‐Based Bioinformatics Analysis

PGK1 expression profiles and corresponding clinical data for HCC were obtained from The Cancer Genome Atlas (TCGA) (https://portal.gdc.cancer.gov). Differential expression of PGK1 between tumor and adjacent normal tissues was analyzed. Overall survival was analyzed using the Kaplan–Meier method with group comparisons by the log‐rank test in the survival package, and survival curves were generated using the survminer package. Immune cell infiltration was estimated using the CIBERSORT algorithm. All statistical analyses were performed in R (version 4.3).

### 4.10 ELISA Assay

The CCL2 concentration in the culture supernatant of tumor cells from different groups was measured using the mouse CCL2 ELISA kit (NBP1‐92659, Novus) in accordance with the manufacturer's instructions.

### 4.11 Multiplex Secretome Analysis

Multiplex secretome analysis of cell supernatants was performed with XMPLEX Mouse 15‐Plex panel(XMPlex03240771) according to the manufacturer's instructions with the assistance of SXM Biotechnology Co., Ltd. (Wuhan, China).

### 4.12 Bone Marrow‐Derived Macrophages (BMDMs) Generation

BMDMs were extracted from the hind limbs of mice and then RPMI 1640 medium supplemented with 25 ng/ml M‐CSF (RP01216, Abclonal), 10% heat‐inactivated FBS, and 1% penicillin/streptomycin. After 7 days of culture, BMDMs were collected for subsequent experiments.

### 4.13 CD8^+^ T Cells Isolation

CD8^+^ T cells were isolated and purified from the spleens of C57BL/6J mice using anti‐CD8 antibody‐conjugated magnetic beads (551516, BD Pharmingen) according to the manufacturer's instructions. Freshly sorted CD8^+^ T cells were cultured in complete RPMI 1640 medium containing 50 U/mL mouse IL‐2 recombinant protein (212‐12, Peprotech), 5 µg/mL purified anti‐mouse CD3 antibody (100302, BioLegend), and 2 µg/mL purified anti‐mouse CD28 antibody (102102, BioLegend) for 3 days.

### 4.14 Transwell Coculture and CFSE Expansion Assay

The detailed information is provided in the Supporting Information.

### 4.15 Migration Assay

The detailed information is provided in the Supporting Information.

### 4.16 Mouse Serum Biochemical Analysis

Mouse serum alanine aminotransferase (ALT), creatinine (Cr), and creatine kinase (CK) levels were measured using an automated biochemical analyzer (BS‐220, Mindray).

### 4.17 Statistical Analysis

Statistical analyses were performed using GraphPad Prism 9 software (La Jolla, CA). In vitro experiments were performed with at least three independent biological replicates. In vivo experiments using animal models and analyses of clinical samples were conducted with a minimum of five independent biological replicates per group. Data are presented as mean ± standard error of the mean (SEM), with each dot in the bar graphs representing an individual biological replicate. Unless otherwise indicated, independent samples were assessed for normality using the Shapiro–Wilk test, and homogeneity of variance was evaluated using Levene's test. For data that met the assumptions of normality and equal variance, statistical differences between two groups were analyzed using a two‐tailed Student's *t*‐test, while comparisons among three or more groups were performed using one‐way analysis of variance (ANOVA) followed by Tukey's post hoc test. For data that did not meet the normality assumption, differences between two groups were assessed using the two‐tailed Mann–Whitney U test, and comparisons among multiple groups were conducted using the Kruskal–Wallis test followed by Dunn's post hoc test with Bonferroni correction. Protein expression levels in tumor and matched adjacent normal tissues were compared using a paired Student's t‐test. Survival analysis was performed using the Kaplan–Meier method, and differences between groups were assessed using the log‐rank test. *p* < 0.05 was considered statistically significant (^*^ *p* < 0.05, ^**^ *p* < 0.01, ^***^ *p* < 0.001, ^****^ *p* < 0.0001), and ns indicates no significance.

## Author Contributions

Xi Liu, Jianpeng Liu, and Zhihao Zhang contributed equally to this work. Xi Liu, Jianpeng Liu, Zhihao Zhang, Yuanxing Liu, and Shusen Zheng designed and supervised the study. Xi Liu, Jianpeng Liu, Zhihao Zhang, Xizhi Yu, Junjie Qian, Linmin Ding, Qinchuan Wu, and Xinjiang Ding performed the experiment. Xi Liu, Jianpeng Liu, Zhe Yang, Xing‐Yu Luo, Rong Su, Xiaobo Yu, and Yiting Qiao performed the acquisition of the data and statistical analysis. All the authors drafted the manuscript. Shengyong Yin, Haiyang Xie, Lin Zhou, Yuanxing Liu, and Shusen Zheng revised the final manuscript. All the authors have approved the final version of the manuscript.

## Conflicts of Interest

The author declares no conflicts of interest.

## Supporting information




**Supporting File**: advs75801‐sup‐0001‐SuppMat.docx.

## Data Availability

The data that support the findings of this study are available from the corresponding author upon reasonable request.
