# Supplementary material for: PGK1 Suppresses CD8+ T Cell‐Mediated Antitumor Immunity Through CCL2/CCR2/Tumor‐Associated Macrophages Axis in Hepatocellular Carcinoma
Source: Adv Sci (Weinh). 2026 May 25:e75801. Online ahead of print. doi: 10.1002/advs.75801 (PMC13336124; doi:10.1002/advs.75801)
Supplement: Supplementary file 1 — Supporting File: advs75801‐sup‐0001‐SuppMat.docx. [file ADVS-9999-e75801-s001.docx]

**Supplementary experimental methods**

**Cell culture and transfection**

The mouse HCC cell line Hepa1-6 was purchased from the American Type Culture Collection (Manassas, VA, USA); the mouse HCC cell line Hep53.4, human HCC cell line HCC-LM3, PLC/PRF/5, and the THP-1 cell line were obtained from Meisen Chinese Tissue Culture Collections (Zhejiang, China). All cell lines were authenticated before use. These cells were routinely cultured in Dulbecco's Modified Eagle Medium (DMEM; Life Technologies, Gaithersburg, MD) or Roswell Park Memorial Institute-1640 (RPMI-1640, Gibco) medium supplemented with 10% fetal bovine serum (FBS) and 1% penicillin/streptomycin solution, and kept in a humidified 5% CO2 incubator at 37 °C.

Short hairpin RNA (shRNA) lentivirus targeted to human PGK1 and mouse PGK1 were respectively constructed by Hanheng Biotechnology (Shanghai, China) and Transheep Biotechnology (Shanghai, China). HCC cells were transduced with PGK1 shRNA lentivirus or negative control lentivirus according to the manufacturer’s instructions. Overexpression plasmids for human PGK1 (pLV3-CMV-hPGK1-3×Flag-Puro) and mouse PGK1(m-Pgk1 pLVX-Puro-Flag), as well as the corresponding vector plasmids, were obtained from RuipuTe Biotechnology (Hangzhou, China).

Small interfering RNA (siRNA) targeting human and mouse β-catenin (siβ-catenin), AKT (siAKT), as well as a scrambled negative control (siNC) were purchased from Sunya Biotechnology (Hangzhou, China). Transfection of plasmids and siRNA was performed using jetPEI (PolyPlus Transfection, France) according to the instructions.

**Mouse tumor models and treatment**

Male C57BL/6J mice (6–8 weeks old) and nude mice (6–8 weeks old) were purchased from Zhejiang Province Experimental Animal Center and Gempharmatech Biotechnology Co., Ltd. To construct an orthotopic HCC model, 1×10^6 HCC cells suspended in 20 μL Matrigel (40187, Yeasen) diluted in DMEM (Matrigel: DMEM, 1:1) were injected into the left liver lobe of mice.

To deplete CD8^+^ T cells in vivo, C57BL/6J orthotopic tumor-bearing mice were injected intraperitoneally with 200 μg of anti-mouse CD8 antibody (BE0061, BioXCell) or isotype antibody (BE0090, BioXCell) starting on day 7 after tumor implantation and subsequently every 3 days. For in vivo suppression of M2-type TAMs, anti-CSF1R (BE0213, BioXCell) was administered intraperitoneally at 300 μg per mouse starting on the day following tumor establishment and was subsequently given every 3 days. To neutralize CCL2 in vivo, 100µg anti‐mouse/human/rat CCL2 antibody (BE0185, BioXCell) was intraperitoneally injected into mice every 3 days from the third day after tumor implantation.

For animal treatment experiments, PGK1 inhibitor NG 52 (HY-15154, MCE; B8515, APExBIO) dissolved in 10% DMSO + corn oil (C7030, Solarbio) or vehicle was administered by gavage with a dosage of 50 mg/kg daily from the second day after tumor implantation. For PD-L1 blockade, each mouse received an intraperitoneal injection of anti–PD-L1 antibody (BE0361, BioXCell) at a dose of 100 μg starting on day 7 and every 3 days thereafter.

In general, mice are euthanized three weeks after tumor implantation, or earlier if the tumor volume exceeds 2000 mm^3^ or predefined humane endpoints are reached, including weight loss, ascites, signs of moribundity, or impaired food and water intake. For survival analysis, animals are monitored until the tumor volume exceeds 2000 mm^3^ or predefined humane endpoints are reached, at which point they are euthanized.

**Single-cell suspension preparation and flow cytometry**

Mouse tumor tissues were dissociated using the Tumor Dissociation Kit (130-096-730, Miltenyi Biotec), according to the manufacturer’s instructions. Briefly, tumors were excised, and necrotic regions were carefully removed before analysis. The remaining tissues were cut into approximately 3 mm pieces, and samples from each tumor were transferred into MACS C Tubes containing RPMI medium supplemented with the enzyme mix. Following dissociation, the cell suspension was passed through a 70-μm cell strainer and subsequently subjected to Percoll density gradient centrifugation (40501ES60, Yeasen) to obtain a single-cell suspension. For intracellular cytokine staining, CD8^+^ T cells were first purified using anti-CD8 antibody-conjugated magnetic beads (551516, BD Pharmingen) and stimulated with Leukocyte Activation Cocktail (550583, BD Pharmingen) in complete RPMI-1640 medium for 6 hours. Fresh tumor tissues from HCC patients were processed and stained following the same protocol as tumor-bearing mice, followed by flow cytometric analysis. Then, samples were stratified into PGK1 high and PGK1 low groups based on RT-PCR–determined PGK1 expression levels, and immune cell infiltration was subsequently compared between the two groups.

**Immunohistochemistry and immunofluorescence**

For the Immunohistochemistry staining, the tissue sections were incubated in EDTA buffer（pH 9.0）at 95°C for 25 minutes for antigen repair. Following the inactivation of endogenous peroxidase with 0.3% hydrogen peroxide and blocking with normal goat serum, sections were incubated with the primary antibody at 4°C overnight. For human tissue sections, antibodies against PGK1 (17811-1-AP, Proteintech) and CD8 (ab101500, Abcam) were used. For mouse tissue sections, antibodies against β-catenin (8480, CST), CCR2 (ab273050, Abcam), and CD206 (ab64693, Abcam) were used. IHC staining scores were quantified using a semi-quantitative scoring system that incorporated both staining intensity and the proportion of positive cells. Staining intensity was graded as 0 (negative), 1 (weak), 2 (moderate), and 3 (strong), while the percentage of positive cells was scored as 0 (<5%), 1 (5–25%), 2 (26–50%), 3 (51–75%), and 4 (>75%). Five representative high-power fields were randomly selected for each sample, and the final IHC scores were calculated as the mean value of the products of the intensity and proportion scores across these fields. For the immunofluorescence assay, the TSA multiplex immunofluorescence staining kit (AFIHC024, Aifang Biological) was used according to the manufacturer’s instructions. And antibodies against PGK1 (17811-1-AP, Proteintech), CD8 (ab101500, Abcam) and CD68 (ab955, Abcam) were used.

**Western blot and Co-immunoprecipitation**

Proteins were extracted with RIPA lysis buffer ( FD008, FUDE) containing 1% protease inhibitor and phosphatase inhibitor (20124ES and 20109ES, Yeasen). Protein electrophoresis was conducted using a 4-20% SDS-containing polyacrylamide (SDS-PAGE) gel. Next, separated proteins were transferred to polyvinylidene fluoride (PVDF) membranes (IPVH00010, Merck Millipore). These membranes were then incubated for at least 12 hours at 4°C with primary antibodies against PGK1(ab199438, Abcam), AKT (9272, CST), phosphorylated-AKT (Ser473, 4060, CST), β-catenin (8480, CST), GSK3-β (22104-1-AP, Proteintech), phosphorylated-GSK3β (Ser9, 67558-1-Ig, Proteintech), CCL2 (23236, CST; 26161-1-AP, Proteintech), β-Tubulin (A12289, Abclonal), and β-actin (AC026, Abclonal). Anti-mouse or anti-rabbit HRP conjugated antibodies were used as secondary antibodies (AS003 and AS014, Abclonal). Target protein bands were visualized using enhanced chemiluminescence reagent (FD8020, FUDE). Quantitative densitometric analysis of all Western blot bands was performed by using ImageJ. Protein expression levels were quantified and normalized to appropriate loading controls (e.g., β-actin or tubulin) to account for differences in sample loading and transfer efficiency. Subsequently, the normalized values were expressed relative to the control group to calculate fold changes in protein expression across experimental conditions.

For the Co-immunoprecipitation assay, proteins of HCC cells transfected with PGK1 overexpression plasmids or vector plasmids were extracted as described above, then Co-IP was performed using anti-Flag magnetic beads (IP) (B26102, Bimake) according to the instructions.

**RNA extraction and quantitative RT-PCR**

RNA extraction was performed with the Total RNA Extraction kit (RK30120, Abclonal). SYBR qPCR Master Mix (Q711-02, Vazyme) was used to perform quantitative real-time PCR after total RNA extraction and complementary DNA reverse transcription (R333-01, Vazyme) according to the manufacturer's instructions. We compared gene expression levels between the groups using the threshold cycle value. Independent experiments were repeated at least three times. Primers were purchased from Sunya (Hangzhou, China). **Table S1** lists the primers used.

**Transwell coculture and CFSE expansion assay**

A noncontact coculture system was established using transwell chambers with a 0.4-μm porous polyester membrane (725122, NEST). For the coculture of CD8^+^ T cells and HCC cells, 2 × 10^5 HCC cells were seeded in the lower compartment. In the upper chamber, either 1 × 10^5 mouse splenic CD8^+^ T cells preactivated in vitro with CD3/CD28 and IL-2 or 1 × 10^5 freshly isolated mouse splenic CD8^+^ T cells labeled with CFSE (423801, BioLegend) were cultured. After 3 days of coculture, CD8^+^ T cells were collected to assess their effector functions, and CFSE dilution was analyzed by flow cytometry to evaluate cell proliferation.

For the coculture of macrophages and HCC cells, 1 × 10^5 BMDMs or THP-1 cells were placed in the upper chamber, and 2 × 10^5 HCC cells were seeded in the lower chamber. In selected intervention or rescue experiments, recombinant mouse CCL2 protein (100 ng/ml, HY-P7764, MCE), recombinant human CCL2 protein (50 ng/ml, HY-P7237, MCE), or anti‐mouse/human CCL2 antibody (5µg/ml, BE0185, BioXCell) was added to the upper chamber as indicated. After 48 hours of coculture, macrophages were collected for flow cytometry analysis.

For the coculture of CD8^+^ T cells and macrophages, BMDMs were first cocultured with HCC cells before the addition of CD8^+^ T cells. 1 × 10^5 BMDMs were placed in the upper chamber, and 2 × 10^5 HCC cells were seeded in the lower chamber. After 48 hours of coculture, recombinant mouse IL-4 protein (214-14, Peprotech) was added to the upper chamber, where indicated to induce M2 polarization of BMDMs for an additional 24 hours. For analysis of CD8^+^ T cells' effector functions, the lower chamber containing HCC cells was removed and replaced with a new lower chamber containing 1 × 10^5 preactivated mouse splenic CD8^+^ T cells, followed by 3 days of coculture before flow cytometric analysis. For proliferation analysis, the lower chamber was replaced with a new lower chamber containing 1 × 10^5 fresh mouse splenic CD8^+^ T cells labeled with CFSE, which were cocultured for 3 days, followed by flow cytometric assessment of proliferation.

**Migration assay**

Transwell migration assay was performed in 24-well transwell chambers with 8 µm pores (3422, Corning). To evaluate CD8^+^ T cells migration, 1 × 10^5 mouse splenic CD8^+^ T cells were added to the upper chamber, and 2 × 10^5 Hep53.4 or Hepa1-6 cells were seeded in the lower chamber. Following incubation (37 °C, 12 hours), the number of CD8^+^ T cells that migrated to the lower chamber was measured by flow cytometry. To evaluate macrophage migration, 1 × 10^5 BMDMs or THP-1-derived macrophages induced by PMA (100 ng/mL, P8139, Sigma) for 24 h were added to the upper chamber, and 2 × 10^5 HCC cells were seeded in the lower chamber. In selected intervention or rescue experiments, recombinant mouse CCL2 protein (100 ng/ml, HY-P7764, MCE), recombinant human CCL2 protein (50 ng/ml, HY-P7237, MCE), or anti‐mouse/human CCL2 antibody (5µg/ml, BE0185, BioXCell) was added to the upper chamber as indicated. After 48 hours of coculture, the transwell membranes were fixed and stained with crystal violet solution, and migrated macrophages were imaged under a microscope and quantified.

**Table S1**

**Primer information**

| Gene | Forward (5′ to 3′) | Reverse (5′ to 3′) |
| --- | --- | --- |
| Mouse-PGK1  Human-PGK1 | ATGTCGCTTTCCAACAAGCTG  CCGCTTTCATGTGGAGGAAGAAG | GCTCCATTGTCCAAGCAGAAT  CTCTGTGAGCAGTGCCAAAAGC |
| Mouse-Actin | GCAGGAGTACGATGAGTCCG | ACGCAGCTCAGTAACAGTCC |
| Human-Actin | TCACCATGGATGATGATATCGC | AATCCTTCTGACCCATGCC |
| Mouse-β-catenn | GTTCGCCTTCATTATGGACTGCC | ATAGCACCCTGTTCCCGCAAAG |
| Human-β-catenn | CACAAGCAGAGTGCTGAAGGTG | GATTCCTGAGAGTCCAAAGACAG |

**Table S2**

**Clinical characteristics of HCC patients with and without response to anti-PD-1/PD-L1 therapy**

|  | non-responder group (n=10) | responder group  (n=10) | *P* value |
| --- | --- | --- | --- |
| Age (year) | 51.10 ± 10.86 | 48.50 ± 13.76 | 0.645 |
| Gender |  |  | 1.000 |
| male | 8 | 9 |  |
| female | 2 | 1 |  |
| BMI (kg/m²) | 22.58 ± 3.48 | 22.87 ± 2.50 | 0.834 |
| Tumor size (cm) |  |  | 1.000 |
| ≤5 | 2 | 3 |  |
| >5 | 8 | 7 |  |
| AFP (ng/mL) |  |  | 0.170 |
| ≤400 | 2 | 6 |  |
| >400 | 8 | 4 |  |
| TNM stage |  |  | 1.000 |
| II/III | 7 | 6 |  |
| IV | 3 | 4 |  |
| MVI |  |  | 0.154 |
| M0 | 1 | 5 |  |
| M1 | 2 | 2 |  |
| M2 | 7 | 3 |  |

BMI, Body Mass Index; MVI, Microvascular Invasion.

**Figure**

**
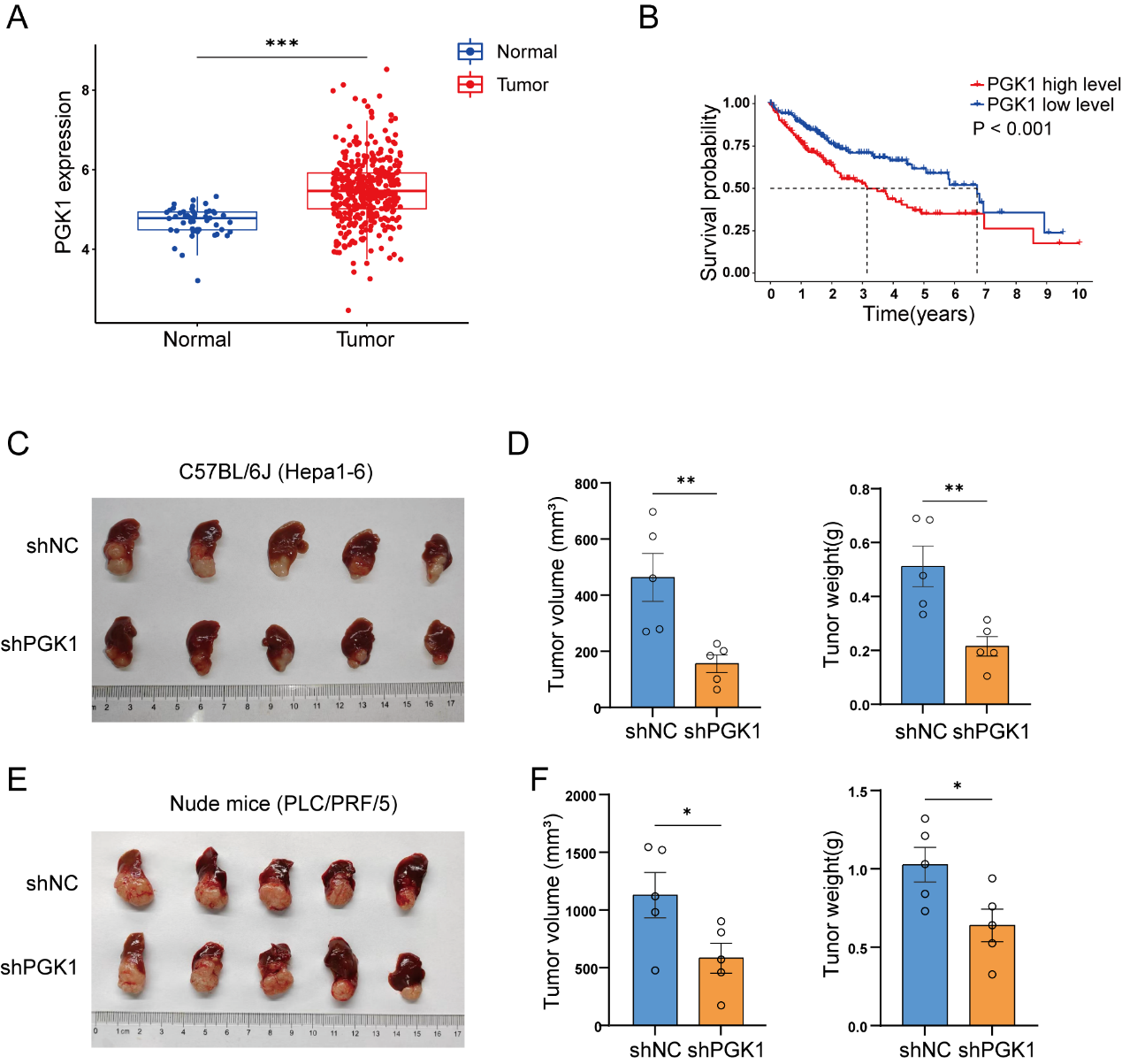
**Figure S1. High expression of PGK1 correlates with poor prognosis in HCC

(A) Comparison of PGK1 expression between HCC tumor tissues and normal tissues based on the TCGA database. (B) Overall survival analysis of HCC patients in the TCGA cohort with high PGK1 expression and low PGK1 expression. (C) Gross images of shPGK1 and shNC Hepa1-6 orthotopic HCC models in C57BL/6J mice (n=5). (D) Tumor volume (left) and tumor weight (right) of shPGK1 and shNC Hepa1-6 orthotopic HCC models in C57BL/6J mice (n=5). (E) Gross images of shPGK1 and shNC PLC/PRF/5 orthotopic HCC models in nude mice (n=5). (F) Tumor volume (left) and tumor weight (right) of shPGK1 and shNC PLC/PRF/5 orthotopic HCC models in nude mice (n=5). Data were presented as mean ± SEM. ns, no significant difference. **P* < 0.05; ***P* < 0.01; ****P* < 0.001; *****P* < 0.0001.


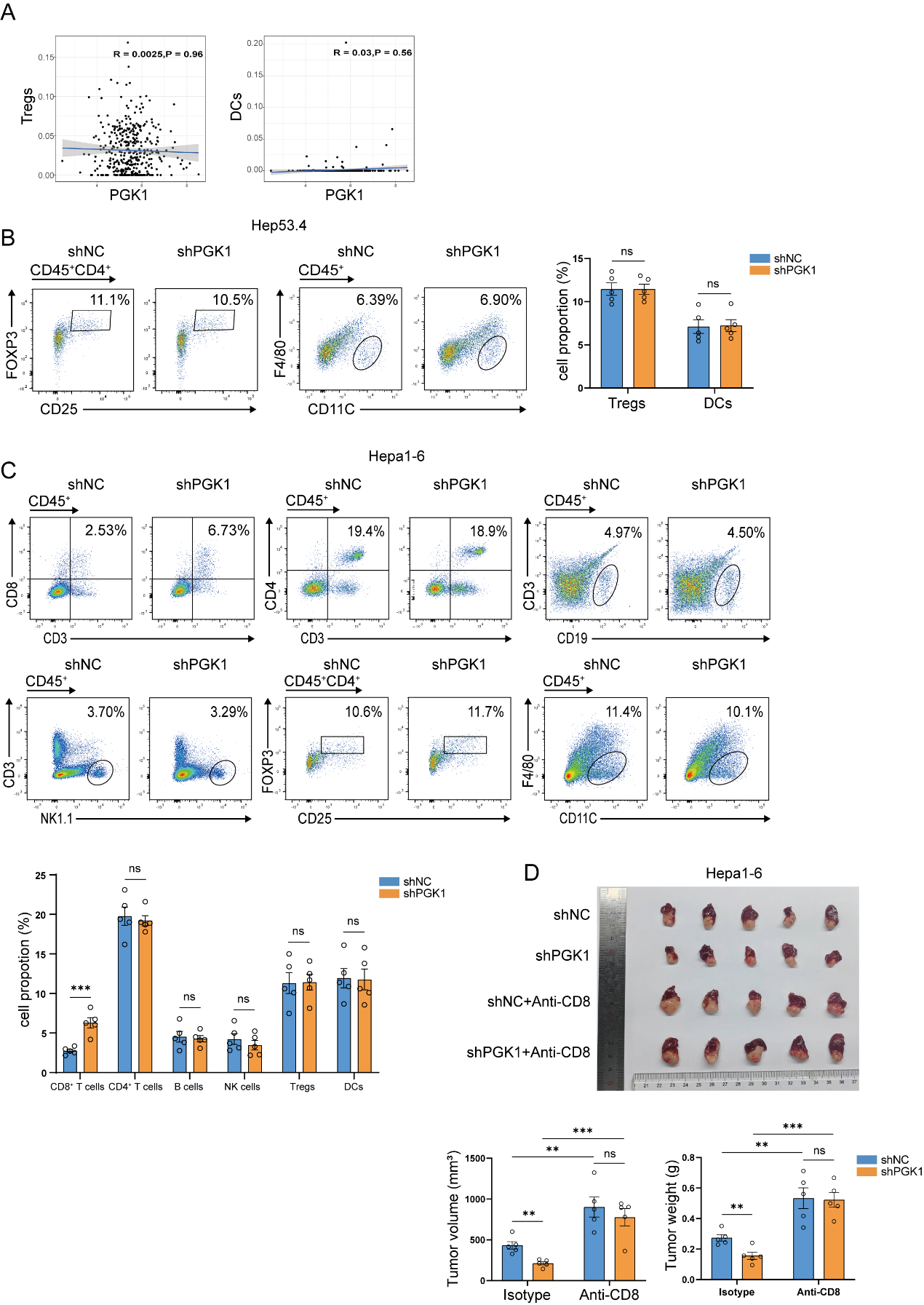


Figure S2. PGK1 promotes tumor progression by inducing immunosuppression through the attenuation of CD8^+^ T cell responses

(A) Analysis of the correlation between PGK1 expression and the infiltration of Tregs and DCs in the TCGA database. (B) Flow cytometry analysis of the infiltration levels of Tregs and DCs in Hep53.4 orthotopic HCC models in C57BL/6J mice (n = 5). (C) Flow cytometry analysis of the infiltration levels of CD8^+^ T cells, CD4^+^ T cells, B cells, NK cells, Tregs, and DCs in Hepa1-6 orthotopic HCC models in C57BL/6J mice (n = 5). (D) Gross images of shPGK1 and shNC Hepa1-6 orthotopic tumors with or without CD8 depletion (upper), and comparison of tumor volume and tumor weight (lower) (n = 5). Tregs, Regulatory T cells. DCs, Dendritic cells. Data were presented as mean ± SEM. ns, no significant difference. **P* < 0.05; ***P* < 0.01; ****P* < 0.001; *****P* < 0.0001.


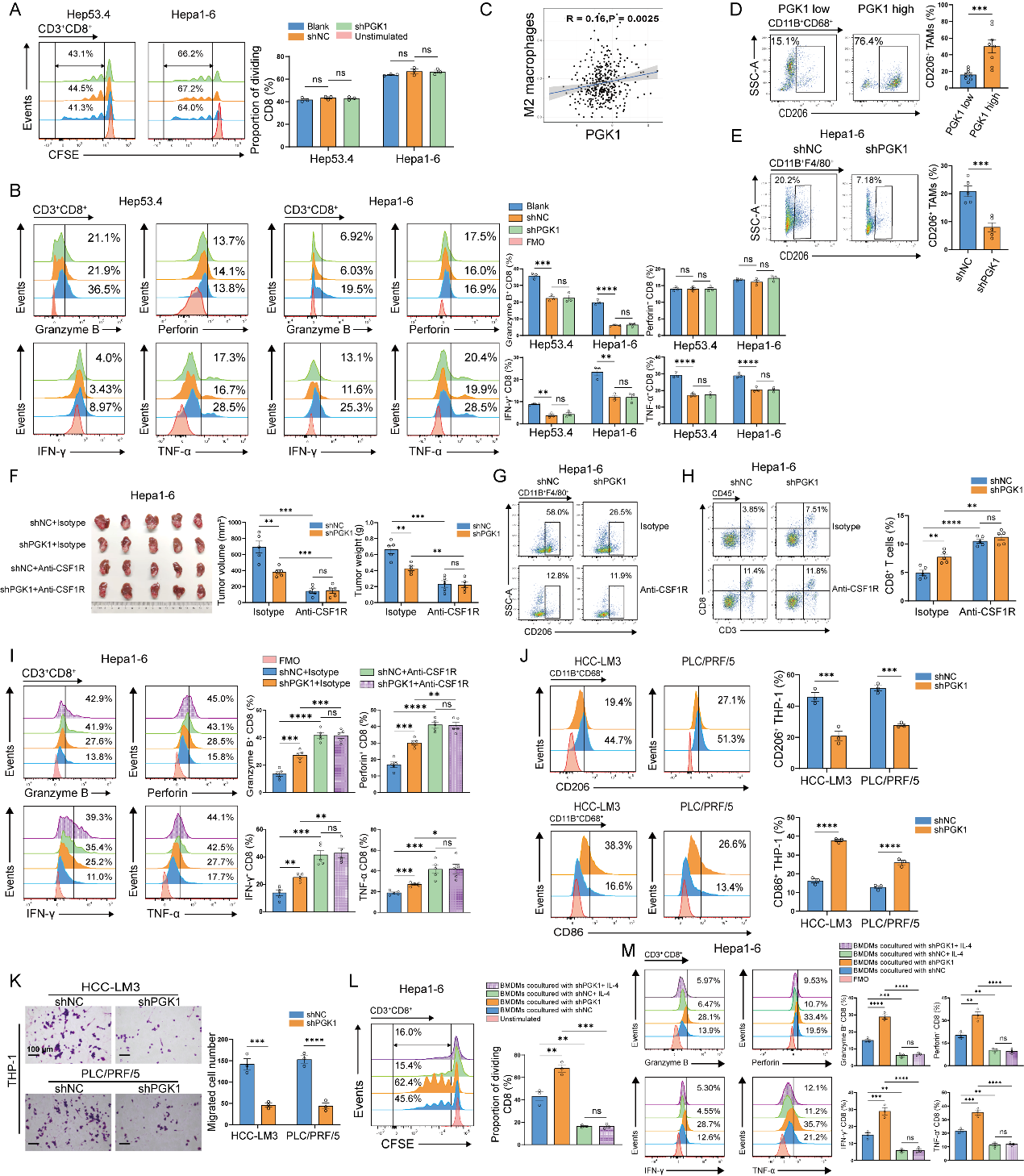


Figure S3. TAMs mediated the suppression effects of PGK1 on CD8^+^ T cells

(A) Comparison of CFSE-labeled mouse splenic CD8⁺ T cells proliferation following coculture with HCC cells transduced with shPGK1 or shNC (n = 3). (B) Effector molecules expression of splenic CD8^+^ T cells cocultured with HCC cells transfected with shPGK1 or shNC (n = 3). (C) Analysis of the correlation between PGK1 expression and M2-type macrophages in the TCGA HCC database. (D) Flow cytometry analysis of the infiltration of M2-type TAMs in PGK1 high expression (n = 8) and low expression samples (n = 8) from HCC patients. (E) Flow cytometry analysis of M2-type TAMs infiltration in shPGK1 and shNC Hepa1-6 orthotopic HCC models (n = 5). (F) Gross images, tumor volume, and tumor weight of shPGK1 and shNC Hepa1-6 orthotopic liver tumors, with or without anti-CSF1R antibody treatment (n = 5). (G) M2-type TAMs infiltration in shPGK1 and shNC Hepa1-6 orthotopic liver tumor models with or without anti-CSF1R antibody treatment. (H) Comparison of CD8^+^ T cells infiltration in shPGK1 and shNC Hepa1-6 orthotopic tumors with or without anti-CSF1R antibody treatment (n = 5). (I) Comparison of Granzyme B^+^, perforin^+^, IFN‐γ^+^, and TNF‐α^+^ CD8^+^ T cells in Hepa1-6 orthotopic HCC models with or without anti-CSF1R antibody treatment (n = 5). (J) The expression of macrophages M2 marker (CD206) and M1 marker (CD86) of THP-1 cocultured with shPGK1 or shNC HCC-LM3 and PLC/PRF/5 cells by flow cytometry (n = 3). (K) Transwell results for THP-1 cocultured with shPGK1 or shNC HCC-LM3 and PLC/PRF/5 cells (n = 3). (L) Comparison of CFSE-labeled mouse splenic CD8⁺ T cells proliferation after coculture with differently treated BMDMs pre-cocultured with Hepa1-6 cells (n = 3). (M) Effector molecules expression (Granzyme B, perforin, IFN‐γ, and TNF‐α) in CD8⁺ T cells cocultured with differently treated BMDMs pre-cocultured with Hepa1-6 cells (n = 3). Data were presented as mean ± SEM. ns, no significant difference. **P* < 0.05; ***P* < 0.01; ****P* < 0.001; *****P* < 0.0001.


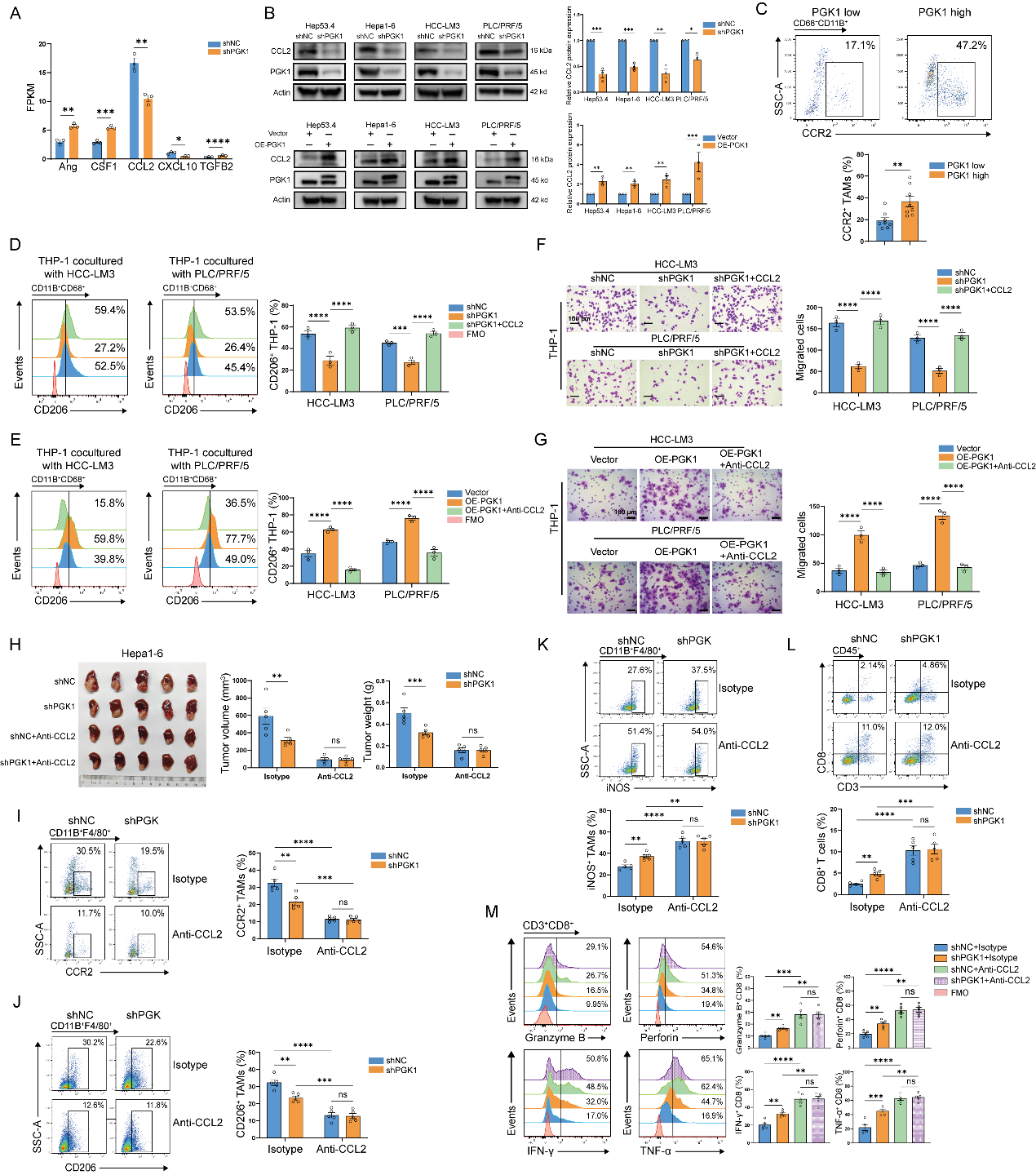


Figure S4. PGK1 enhances the recruitment and M2 polarization of TAMs in a CCL2-dependent manner

(A) FPKM values of differentially expressed cytokine genes identified by transcriptome sequencing (n = 3). (B) Western blot analysis showing CCL2 expression in PGK1-knockdown (upper) and PGK1-overexpressing HCC cells (lower) (n = 3). (C) Flow cytometry analysis of the infiltration of CCR2^+^ TAMs in PGK1 high expression (n = 8) and low expression (n = 8) samples from HCC patients. (D) Expression of the M2-type macrophage marker CD206 in THP-1 cocultured with the indicated HCC cells, with or without supplementation of recombinant CCL2 protein (50 ng/mL) (n = 3). (E) Expression of the M2-type macrophage marker CD206 in THP-1 cocultured with the indicated HCC cells, with or without supplementation of anti-CCL2 antibody (5 μg/mL) (n = 3). (F) Transwell results for THP-1 cocultured with the indicated HCC cells, with or without supplementation of recombinant CCL2 protein (50 ng/mL) (n = 3). (G) Transwell results for THP-1 cocultured with the indicated HCC cells, with or without supplementation of anti-CCL2 antibody (5 μg/mL) (n = 3). (H) Gross images, tumor volume, and tumor weight of shPGK1 and shNC Hepa1-6 orthotopic tumors in C57BL/6J mice, with or without anti-CCL2 antibody treatment (n = 5). (I) Flow cytometry analysis of CCR2^+^ TAMs infiltration in Hepa1-6 orthotopic HCC models with or without anti-CCL2 antibody treatment (n = 5). (J) Comparison of M2-type TAMs infiltration in Hepa1-6 orthotopic HCC models with or without anti-CCL2 antibody treatment (n = 5). (K) Comparison of M1-type TAMs (iNOS^+^ TAMs) infiltration in Hepa1-6 orthotopic liver tumor models with or without anti-CCL2 antibody treatment (n = 5). (L) Comparison of CD8^+^ T cells infiltration in Hepa1-6 orthotopic HCC models with or without anti-CCL2 antibody treatment (n = 5). (M) Comparison of Granzyme B^+^, perforin^+^, IFN‐γ^+^, and TNF‐α^+^ CD8^+^ T cells in Hepa1-6 orthotopic HCC models with or without anti-CCL2 antibody treatment (n = 5). Data were presented as mean ± SEM. ns, no significant difference. **P* < 0.05; ***P* < 0.01; ****P* < 0.001; *****P* < 0.0001.


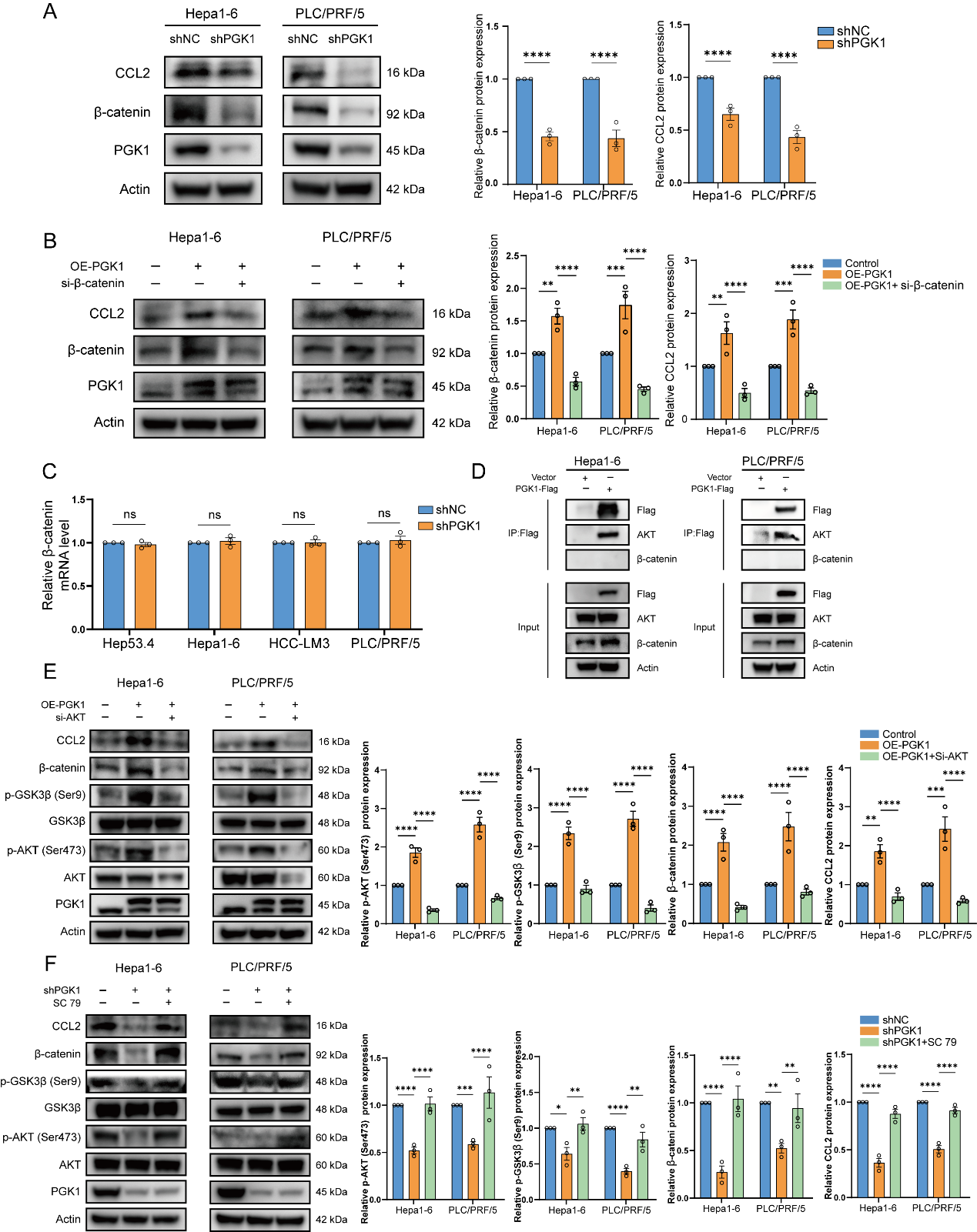


Figure S5. PGK1 upregulates CCL2 expression by activating AKT/GSK-3β/β-catenin signaling pathway in HCC cells

(A) Western blot analysis of β-catenin and CCL2 expression in PGK1 knockdown and control HCC cells (n = 3). (B) Western blot analysis of CCL2 expression in PGK1-overexpressing HCC cells with or without pre-established β-catenin knockdown (n = 3). (C) Relative β-catenin mRNA levels in PGK1 knockdown HCC cells compared to the control group (n = 3). (D) Co-IP assays showing whether β-catenin and AKT interact with PGK1 in HCC cells. (E) Western blot analysis of p-AKT (Ser473), p-GSK3β (Ser9), β-catenin, and CCL2 expression in PGK1-overexpressing HCC cells with or without pre-established AKT knockdown (n = 3). (F) Western blot analysis of p-AKT (Ser473), p-GSK3β (Ser9), β-catenin, and CCL2 in PGK1 knockdown HCC cells treated with or without the AKT activator SC79 (10 μM) (n = 3). Data were presented as mean ± SEM. ns, no significant difference. **P* < 0.05; ***P* < 0.01; ****P* < 0.001; *****P* < 0.0001.


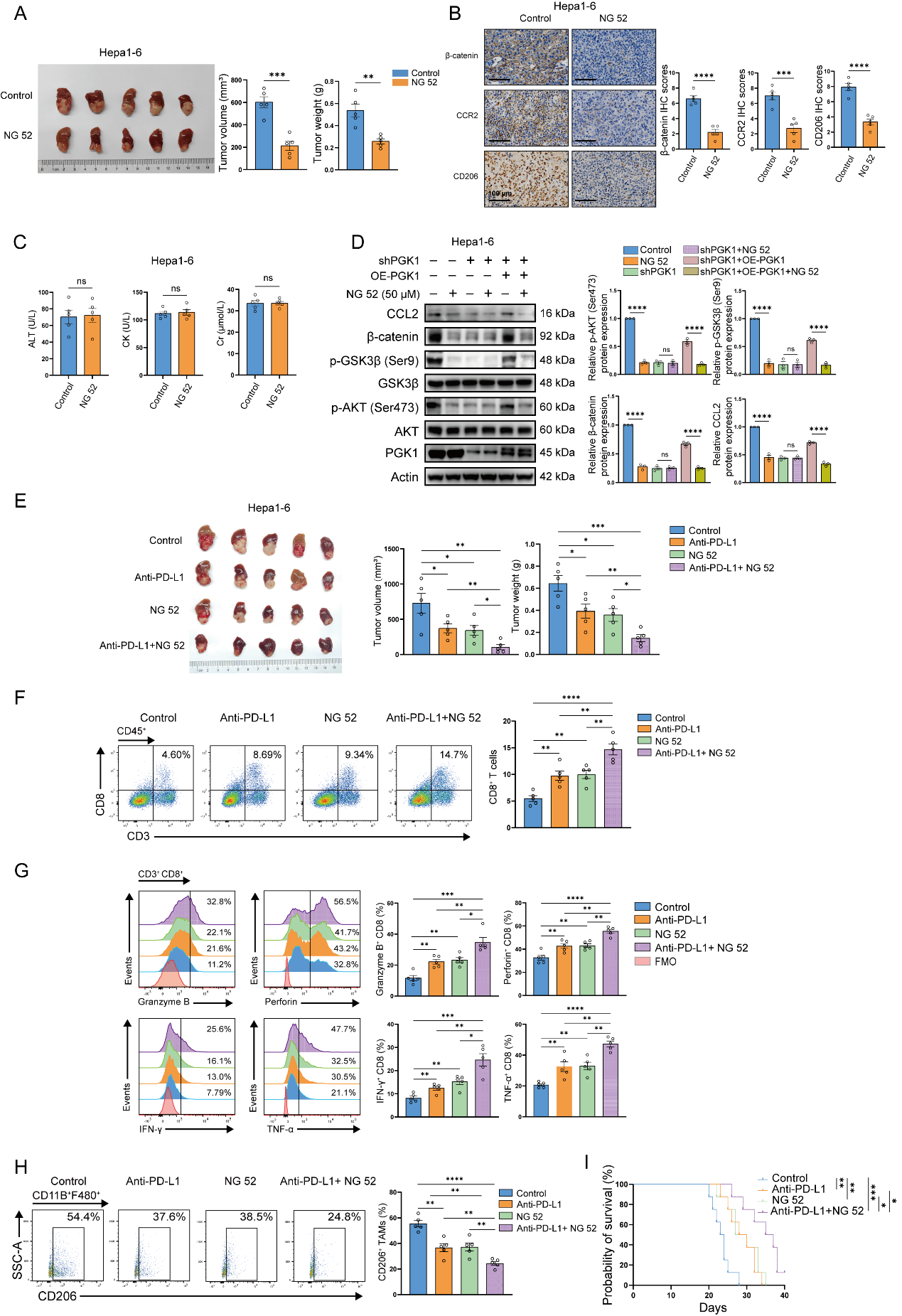


Figure S6. Pharmacological targeting of PGK1 improves the response of HCC to anti-PD-L1 therapy

(A) Gross images, tumor volume, and tumor weight of Hepa1-6 orthotopic HCC models in C57BL/6J mice, with or without PGK1 inhibitor NG 52 treatment (n = 5). (B) Representative IHC staining images (400x magnification) and comparison of IHC scores for β-catenin, CCR2, and CD206 in Hepa1-6 orthotopic tumors, with or without NG 52 treatment (n = 5). (C) Serum ALT, CK, and Cr levels in mice with Hepa1-6 orthotopic tumors, with or without NG 52 treatment (n = 5). (D) Western blot analysis of p-AKT (Ser473), p-GSK3β (Ser9), β-catenin, and CCL2 in Hepa1-6 cells under different treatments (n = 3). (E) Gross images, tumor volume, and tumor weight of Hepa1-6 orthotopic tumors treated with anti–PD-L1 antibody, NG 52, or the combination (n = 5). (F) Flow cytometry analysis of CD8⁺ T cells infiltration in Hepa1-6 orthotopic liver tumor models subjected to different treatments (n = 5). (G) Comparison of Granzyme B^+^, perforin^+^, IFN‐γ^+^, and TNF‐α^+^ CD8^+^ T cells in Hepa1-6 orthotopic HCC models subjected to different treatments (n = 5). (H) Infiltration of M2-type TAMs in Hepa1-6 orthotopic HCC models with different treatments (n = 5). (I) Survival of Hepa1-6 orthotopic HCC models with various treatments (n = 8). HR and 95% CI: Anti–PD-L1 vs. Control, 0.1544 (0.0417–0.5717); NG 52 vs. Control, 0.1651 (0.0450–0.6050); Combination vs. Control, 0.0539 (0.0122–0.2371); Combination vs. Anti–PD-L1, 0.1898 (0.0521–0.6903); Combination vs. NG 52, 0.2488 (0.0723–0.8558). ALT, alanine aminotransferase. Cr, creatinine. CK, creatine kinase. Data were presented as mean ± SEM. ns, no significant difference. **P* < 0.05; ***P* < 0.01; ****P* < 0.001; *****P* < 0.0001.
